# Visual exploration of omnidirectional panoramic scenes

**DOI:** 10.1167/jov.20.7.23

**Published:** 2020-07-21

**Authors:** Walter F. Bischof, Nicola C. Anderson, Michael T. Doswell, Alan Kingstone

**Affiliations:** University of British Columbia, Vancouver, Canada

**Keywords:** eye movements, head movements, gaze-head relationship, omnidirectional panoramic scenes

## Abstract

How do we explore the visual environment around us, and how are head and eye movements coordinated during our exploration? To investigate this question, we had observers look at omnidirectional panoramic scenes, composed of both landscape and fractal images, using a virtual reality viewer while their eye and head movements were tracked. We analyzed the spatial distribution of eye fixations and the distribution of saccade directions and the spatial distribution of head positions and the distribution of head shifts, as well as the relation between eye and head movements. The results show that, for landscape scenes, eye and head behavior best fit the allocentric frame defined by the scene horizon, especially when head tilt (i.e., head rotation around the view axis) is considered. For fractal scenes, which have an isotropic texture, eye and head movements were executed primarily along the cardinal directions in world coordinates. The results also show that eye and head movements are closely linked in space and time in a complementary way, with stimulus-driven eye movements predominantly leading the head movements. Our study is the first to systematically examine eye and head movements in a panoramic virtual reality environment, and the results demonstrate that a virtual reality environment constitutes a powerful and informative research alternative to traditional methods for investigating looking behavior.

## Visual exploration of omnidirectional panoramic scenes

Humans explore the visual environment by moving their eyes several times per second to sample different locations. This is necessitated by the variable-resolution characteristics of the retina and thus the need to project different locations of the environment onto the high-resolution fovea for processing during each fixation. Evidence from many studies on the characteristics of fixations and saccades suggests that these locations are not randomly selected (e.g., Buswell, 1935; Yarbus, 1967). Fixations occur significantly more often near the center of the visual field (central fixation bias, Tatler, 2007), on scene parts that are rated as informative rather than redundant (Mackworth & Morandi, 1967; Underwood & Foulsham, 2006), on items that convey social information, such as faces of people (e.g., Birmingham, Bischof, & Kingstone, 2008, 2009; Yarbus, 1967), or on regions of high contrast in low-level image features (e.g., Parkhurst, Law, & Niebur, 2002).

The nonrandom nature of selecting fixation positions is also evident from the characteristics of saccades. Several studies have shown systematic tendencies in saccade directions, with saccades executed preferably in the cardinal directions rather than the obliques (Foulsham, Kingstone, & Underwood, 2008; Foulsham & Kingstone, 2010). The bias of saccade directions could be due to oculomotor factors, namely, the dominance of the muscle or neural apparatus, which preferentially triggers horizontal shifts of the eyes, regardless of the stimulus being viewed. The bias could also be a consequence of the distribution of salient features in pictures and natural scenes. Furthermore, a learned account could predict a horizontal bias based on our experience with pictures and the environment that is acquired over time and initiated top-down, to reflect one's goals and expectations. Finally, the biases in saccade direction could be display-specific, an artefact of laboratory-based eye tracking studies that present scenes on a computer monitor.

To investigate the roots of these saccade biases, Foulsham et al. investigated saccadic behavior while viewing images rotated to various extents within frames that remained constant or were also rotated (Foulsham et al., 2008; Underwood, Foulsham & Humphrey, 2009; Foulsham & Kingstone, 2010). For instance, Foulsham et al. presented natural images that were rotated by 0°, 45°, 90°, 135°, or 180°. The results showed that saccades in the horizon direction of the images were more frequent and larger in amplitude, independent of the image orientation. For images with indoor scenes (which have an increased presence of vertical edges), the saccades were biased in the cardinal directions (left–right and up–down), again with respect to the image “horizon.” Foulsham et al. concluded that these results rule out a simple oculomotor explanation of saccade generation and favored a combination of image-based and frame-based accounts: First, the distribution of image features (edges or salient locations) may guide eye movements in a bottom-up (stimulus-driven) manner toward the image horizon. Second, the gist of the images (Potter, Staub, & O'Connor, 2004; Torralba, 2003) conveyed by the low spatial frequencies of the images may guide the eyes in a top-down manner towards the horizon where the most informative image features are likely to be located. Finally, the increased presence of vertical and horizontal edges in natural images (Coppola, Purves, McCoy, & Purves, 1998) may be the cause for the increased presence of saccades in the cardinal, but not oblique, directions in images.

The frame that the stimuli are presented in also seems to be of critical importance. For example, Foulsham et al. (2008) presented landscape and interior scenes that were rotated at various angles, but within a constant square frame. They found that the dominant saccade direction followed the orientation of the scene (e.g., the horizon for landscape scenes), although this was less pronounced for interior scenes. In contrast, when the images were presented within a circular frame, Foulsham and Kingstone (2010) found that the predominant saccade direction bias was orthogonal to, rather than parallel to, the image horizon. It was unclear why this difference occurred, but extraneous factors such as the rectangular shape of the monitor, which are normally wider than they are high, were identified as playing a key role. This possibility has recently been supported by a systematic study of the issue by Anderson, Bischof, Foulsham, and Kingstone (in press).

**Figure 1.**
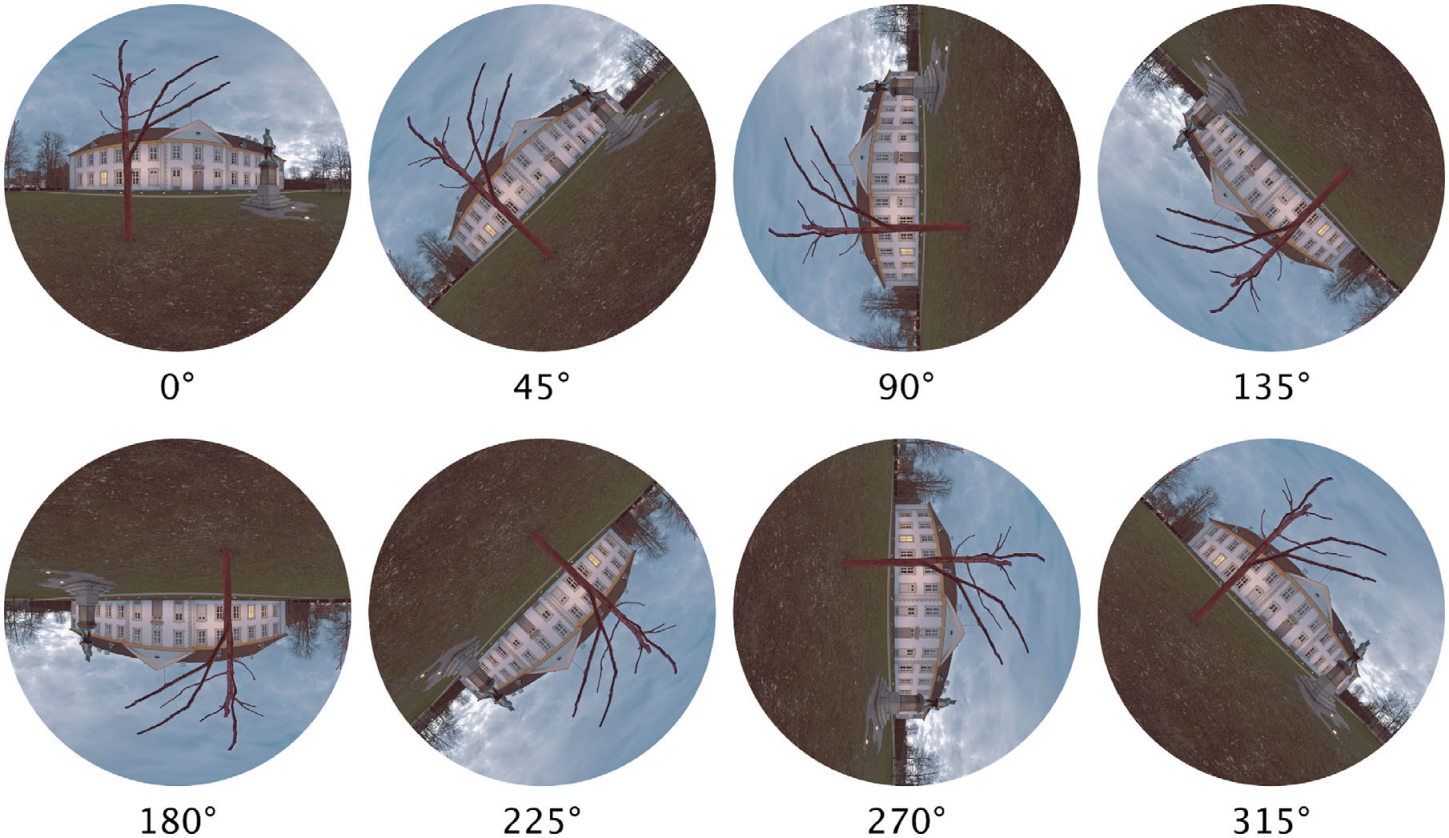
Sketch of the 360° panoramas rotated counterclockwise by 0°, 45°, …, 315°. Note that this simple illustration shows only a small portion of the display that surrounded and immersed participants.

To control for these possible limitations, we compared the eye movements of people immersed in a virtual environment with 360° panoramic scenes while their head was unrestrained. We asked whether people would distribute their gaze in the same way as past studies, and what role, if any, the head plays in the visual exploration. This design has several advantages over previous studies. First, following Foulsham et al., we rotated the panoramas around the view axis to study the effect of image rotation on saccade directions (Figure 1), but our design allowed to study these effects more precisely by eliminating any reference frames defined by the stimulus edge or the monitor frame. Second, the view of the environment was not preselected by the experimenter, but selected by the participant because their head was free to move. This design can thus shed light on both how one explores and inspects the visual environment. Third, the design permitted us to study the relation between, and the coordination of eye and head movements under general, but well controlled conditions. These advantages are discussed in greater detail elsewhere in this article.

To date, the vast majority of studies have measured eye movements with the observer's head immobilized, usually by a chinrest, but more extreme measures, such as a bite-bar, were once routine. On the one hand, immobilizing the head not only permits accurate eye movement measurements, but it also enables one to study oculomotor processes in isolation, uninfluenced by head or body movements. On the other hand, this approach means that all the visual information displayed to the observer has been pre-selected by the experimenter, that is, the observer is a passive recipient of the visual information. To the extent that researchers wish to understand how people explore and inspect the visual information that surrounds them, and not just how people make eye movements to pictures that are presented to them, these limitations are potentially profound.

The idea that laboratory-based, two-dimensional monitor-based experiments do not generalize to real life is not new (e.g., Land & Hayhoe, 2001; Hayhoe & Ballard, 2005; Kingstone, Smilek, & Eastwood, 2008; Tatler, Hayhoe, Land, & Ballard, 2011). In real life, people turn their heads and bodies to acquire and view new information that is currently unavailable. For example, when we hear a loud noise behind us on the street, we turn our heads to explore and inspect the source of the noise. In other words, the act of selecting a new view by turning one's body and head, and the process of examining the information that is delivered to the eyes by a head turn, are complimentary processes. Many studies have provided evidence for a close interplay between gaze and head movements, both in the real world and in virtual reality (VR). In studies of navigation through natural environments, Foulsham, Walker, and Kingstone (2011) analyzed the visual behavior of participants as they walked across a university campus wearing a mobile eye tracker and found that locations around the horizon were selected with head movements, and eye movements were centralized within that head-centered visual field. Similarly, Einhäuser et al. (2007) studied eye–head coordination in natural exploration and provided evidence for synergistic eye and head movements in adjusting gaze during navigation. The close interplay between head and gaze movements is also evident from studies that analyzed gaze and head movements during interactions with objects in the environment, for example, when making a cup of tea or a sandwich (Land, Mennie, & Rusted, 1999; Land & Hayhoe, 2001; Land & Tatler, 2009). These studies provide evidence that gaze plays specific roles in these activities, including, for example, locating objects for future use, establishing a target direction for movements, guiding movements, and checking that particular conditions have been met (Land & Hayhoe, 2001). They have also established the important role of internal rewards in guiding eye and body movements (Hayhoe & Ballard, 2005).

The present study attempts to build a bridge between these two categories of studies. That is, the studies that investigate the characteristics of eye movements on two-dimensional monitors with head movements of participants discouraged and often constrained; and the studies that investigate the coordination of gaze and head movements in complex, natural, or virtual environments. When the head is free to move, changes in the line of sight involve simultaneous saccadic eye and head movements. In this situation, the rules defining head-restrained saccadic eye movement have to be altered (Bizzi, Kalil, & Tagliasco, 1971; Freedman, 2008). With attention shifts to sudden, bottom-up cues, for example, the line of sight, is adjusted quickly through a saccadic eye movement, followed by the slower head movement combined with eye movement to keep the relevant stimulus projected on the fovea. With top-down control of the line of sight, the head may move first to select a newly centered visual field, followed by the eyes (Doshi & Trivedi, 2012). Taken together, adjustments of gaze, that is, the line of sight, are achieved through a combination of eye and head movements, both with different functions in visual exploration and with different temporal characteristics. As Solman, Foulsham, and Kingstone (2017) showed using gaze- and head-contingent displays, eye movements are more likely to move in the direction of visible information, whereas head movements are complementary, moving primarily in a direction to reveal new information. Moreover, different cognitive effects of head and eye movements were found by Solman and Kingstone (2014). They discovered greater use of memory in head-contingent search compared with gaze-contingent search, which they attribute to the difference in energetic cost of turning the head to orient attention (e.g., having worked hard to turn one's head to see an item, it is prudent to commit it to memory).

**Figure 2.**
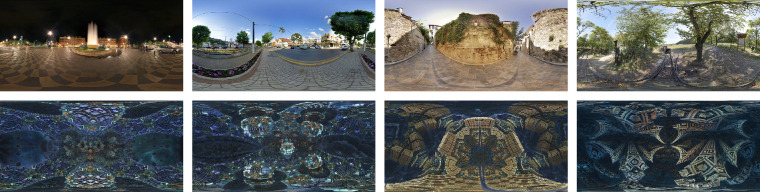
Samples of the panoramic scenes used in the present experiment. Landscape scenes are shown in the top row, fractal scenes in the bottom row.

In the present study, participants were immersed in a virtual environment containing panoramic scenes with landscape scenes and fractal scenes. Most of the landscape scenes have a fairly well-defined horizon, which separates the top half (sky) from the bottom half (ground; Figure 2a through 2d). To investigate the effect of image content on visual exploration (e.g., the effect of image horizon), we also used fractal panoramas (Figure 2e through 2h). The landscape and fractal images contained similar spatial frequency variations, in the sense that the spectra for both scene types followed approximately a 1/f distribution. The fractal panoramas lack any clear semantically meaningful items including horizons, but, as illustrated in Figure 2e through 2h, many show left–right or up–down symmetry or both, which may potentially have an effect on the visual exploration patterns. In addition, all panoramas were rotated around the view axis, as shown in Figure 1 and described in greater detail elsewhere in this article.

**Figure 3.**
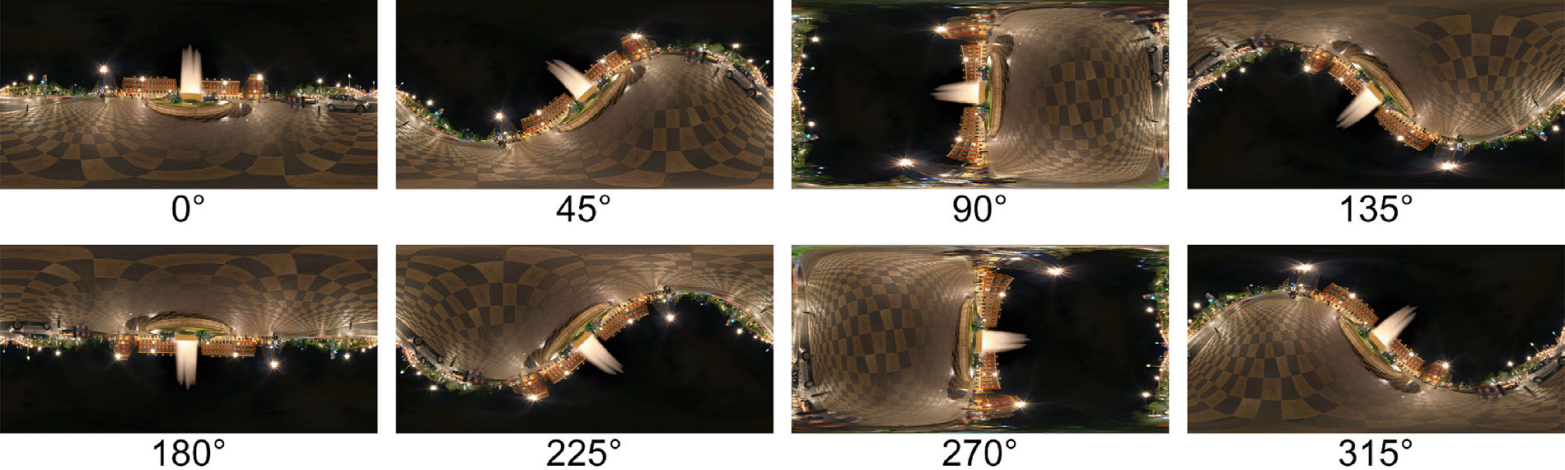
Panoramas projected on an equirectangular (or equidistant) map, for rotations 0°, 45°, …, 315°. Each map is shown for longitudes in the range of −180° to 180° and latitudes in the range of −90° to 90°. Note that, for panorama rotations 90° and 270°, the image along the 0° meridian is continued on the 180° meridian, leading to the appearance of a “wrap-around” of the panoramas.

The panoramas were projected on the inside of a virtual sphere, with the participants’ head positioned in the middle. The panoramas are defined in world coordinates, with longitudes in the range of −180° to 180° (from west to east) and latitudes in the range of −90° to 90° (from the south pole to the north pole). Panorama images and data are presented on a flat projection, an equirectangular (or equidistant) projection (Wikipedia, 2019). This projection maps meridians into to vertical straight lines of constant spacing, introducing strong distortions near the poles compared with the equator (the horizon area), hence data in the polar regions have to be interpreted carefully. The panoramas were rotated in steps of 45° around a line defined by the two points with longitudes of 0° and 180°and a latitude of 0°. The projection of the rotated panorama in Figure 2a is illustrated in Figure 3.

### Definition of terms and data analysis

There is substantial confusion in the eye movement field regarding the definition of fixations and saccades, in particular when eye movements are combined with free head movements (see e.g., Hessels, Niehorster, Nyström, Andersson, & Hooge, 2018). In our study, participants were seated inside a virtual sphere and looked at the panoramic scenes with free head movements from approximately the center of the sphere. For many of the measures below, we chose to define them with respect to the stimulus sphere, to allow for the expression of eye and head measures, and their comparison, with respect to a common coordinate frame. In this context, we now define terms related to gaze, head, and eyes.

The main gaze analyses refer to the gaze vector in a world coordinate frame, which combines the head direction and the gaze direction within the head. A gaze hit point, what we call a “gaze point” for short, is defined as the intersection of the gaze vector with the virtual sphere on which the panoramas are projected. It is defined in world coordinates, with longitudes in the range of −180° to 180° and latitudes in the range of −90° to 90°. Torsional eye movements (or ocular counter-roll; e.g., Schmid-Priscoveanu, Straumann, & Kori, 2000; Chandrakumar, Blakeman, Goltz, Sharpe, & Wong, 2011; Schor, 2011) were not measured by our system. Fixations are defined as stable gaze points and are extracted from the unfiltered gaze data using the Identification by dispersion–threshold algorithm (Salvucci & Goldberg, 2000; Blignaut, 2009; Komogortsev, Gobert, Jayarathna, Koh, & Gowda, 2010), applying a minimum duration of 80 ms and a maximum dispersion threshold of 3° great circle distance. Saccades are defined as angular differences between successive fixations (Appendix A).

With respect to the head, we define head pitch (achieved through neck extension and flexion), head yaw (achieved through lateral neck rotation), and head roll (achieved through lateral bending of the neck). The head hit point, or “head point,” is defined as the intersection of the vector pointing forward from the face with the virtual sphere and is also defined in world coordinates, that is, longitude and latitude of the panorama. Head roll is measured counterclockwise from the vertical head orientation. Head movements with the VR viewer are usually very smooth and do not permit identifying stable points from the raw data. For this reason, we define a head fixation as the average head point during a fixation. Head shifts are defined as the angular differences between successive head fixations (Appendix A).

Finally, we define gaze-in-head as the gaze direction in a head-centered coordinate system. The eye hit point, or eye point, is defined as the difference between gaze point and head point and is also expressed in world coordinates, that is, in longitude and latitude, with the origin (longitude and latitude equal to 0°) at the head point.

By choosing to define the head position relative to stable gaze positions, we acknowledge that the contribution of the vestibulo-ocular reflex (VOR; Barnes, 1979; Laurutis & Robinson 1986) is not taken into account. Almost certainly, during a gaze fixation, the VOR could be acting to stabilize the gaze position during a head movement (Appendix C), and in our definition of a head fixation, these head movements would be averaged.

## Methods

### Participants

Eighteen participants (ages 18–37, *M* = 21.3 years, 12 female) from the University of British Columbia participated in this experiment for course credit. All reported normal or corrected-to-normal vision. Participants provided informed consent before participation, and the study was approved by the ethics board of the University of British Columbia (H10-00527).

### Apparatus

The stimuli were presented on an HTC Vive Virtual Reality headset equipped with an SMI eye tracker (SensoMotoric, 2017), controlled by a custom-built desktop computer (PC with Intel i7-8700K CPU @ 3.70 GHz, 32 GB RAM, Nvidia GeForce GTX 1080 Ti, 1TB Samsung SSD). The HTC Vive headset (Vive model 2PU6100) has a display resolution of 1080 × 1200 pixels, a horizontal field of view of approximately 110°, a vertical field of view of approximately 113°, a refresh rate of 90 Hz, and a weight of 635 g.

The SMI eye tracker recorded eye movements at 250 Hz within the full field of view (110°). The manufacturer-claimed accuracy is typically 0.2°. Our experience with the system gives us no reason to question this figure; however, note also that the results of the present study do not depend on this level of accuracy. To measure head movements, two infrared base stations situated in opposite corners of the room tracked the location of the HTC Vive headset at 90 Hz. According to Niehorster, Li, and Lappe (2017), the orientation precision of the Vive headset is on the order of 0.01° for a nonmoving observer, as was the case in the present study. The position and orientation of the headset were fed unfiltered into our analyses. Participants were seated in a swivel chair in the center of the room and held a keyboard to provide input throughout the experiment.

The virtual space for stimulus presentation was created in Unity (version 2017.3.1; Technologies, 2015). Stimulus presentation and the recording of gaze and head data were driven by Unity using an SMI plugin (version 1.0.2) under experimental control of a custom C# program. Data analyses was done using Matlab R2019a (Mathworks, 2019), and statistical analyses were done using Stata 15.1 (Stata, 2019).

### Stimuli

The virtual space was created using Unity (Technologies, 2015) and consisted of a sphere around the participant, onto which different omnidirectional panoramic scenes were projected, such that the participant appeared to be immersed in the scenes. The scenes consisted of 160 full 360° panoramic images with 80 landscape scenes and 80 computer-generated 3D fractal scenes. The landscape scenes were taken from the SUN360 Panorama Database (Xiao, Ehinger, Oliva, & Torralba, 2012). The computer-generated fractal images were requested from the Fractal Canyon website (Schizo604, 2010). All images had a resolution of 4096 × 2160 pixels. The scenes were presented in eight rotations, rotated counter-clockwise about a horizontal axis (the *z*-axis of the virtual space) by 0°, 45°, 90°, 135°, 180°, 225°, 270°, and 315° (see Figure 1). The SMI system permits stereoscopic displays and the analysis of binocular eye movements, but we decided against using it for the present study. For this reason, the scenes appeared “painted” inside the virtual sphere with binocular or motion depth cues absent.

### Procedure

At the start of the experiment, participants were familiarized with the VR equipment and instructed to remember the presented scenes for a subsequent memory test. They were handed a keyboard and instructed to use the enter key for starting a stimulus presentation. The experiment proceeded in two phases. In the first phase, participants viewed 160 scenes with the instruction to remember them for later testing. In the second phase, participants were presented with 10 old and 10 new, unrotated scenes, and had to indicate whether they had seen the scenes before. The purpose of the memory procedure was merely to ensure that participants engaged in scanning the panoramas, and the delivery of the test was done to ensure that, if participants talked to one another outside the study, then future participants would know that the memory instruction was authentic, and not merely a deception.

At the beginning of the experiment and after every 20 trials, the eye tracker was calibrated using a 5-point calibration test, in which the participants had to follow with their eyes a white dot with a red center as it moved to five locations in visible space, that is, from the center of the head to four equally spaced dots in random sequence, each dot appearing on the corner of an imaginary square, then back to center. Calibration was yoked to head position, such that if a participant moved their head, the fixation point moved correspondingly. Thus, we asked participants to keep their head still during calibration. After a successful calibration, the experimental procedure continued, otherwise the calibration was repeated until it was successful. According to the manufacturer, not only the eye tracking data while fixating on a static position, but also the transition phases in between were used for calibration.

In the initial viewing phase, each trial began with a fixation cross in the screen center. This allowed the experimenter to monitor the accuracy of the eye tracker. Participants pressed the enter key to initiate a new trial. Each scene was presented for 10 seconds, long enough to allow slower head movements during the stimulus presentation. There were 160 trials, 80 with fractal scenes and 80 with landscape scenes Thus, there were 80 unique landscape and 80 unique fractal scenes, and these scenes were shown in one of eight different orientations, resulting in 10 scenes per orientation and scene type. The scene orientations were balanced across participants to ensure that each scene was presented in every orientation. This phase of the experiment took approximately 20 minutes.

In the recognition phase, the same procedure was followed, except that, at the end of each trial, participants had to indicate on the keyboard whether they had seen the scene before or whether it was a new scene. Forty scenes were presented, 10 old landscape scenes, 10 old fractal scenes, 10 new landscape scenes, and 10 new fractal scenes, each one being presented for 10 seconds. This phase of the experiment took approximately 10 minutes.

The gaze direction vector was recorded at 250 Hz while head position (i.e., the position of the headset) and head orientation were recorded at a rate of approximately 70 Hz rate, with the exact timing being dependent on the Unity system (Technologies, 2015). Head position and orientation were linearly interpolated to 250 Hz, and a gaze vector was extracted by combining head and eye direction. The gaze and head positions were transformed into longitude and latitude values in the spherical panorama, with longitudes in the range of −180° to 180° and latitudes in the range of −90° to 90°.

## Results

### Recognition performance

In the recognition phase, 40 scenes were presented, 10 old landscape scenes, 10 old fractal scenes, 10 new landscape scenes, and 10 new fractal scenes, each one being presented for 10 seconds. Recognition performance is shown in Table 1, which indicates that participants were on task and better at recognizing landscape scenes than fractals, *t*(34) = 6.21, *p* < 0.001, *d* = 2.07. Binomial tests showed that 13 (of 18) participants recognized the landscape scenes above chance level, but only two (of 18) recognized the fractal scenes above chance level. Further analyses failed to relate the level of recognition performance to the gaze and head analyses reported below.

**Table 1.** Recognition performance of landscape and fractal panoramas (in percent correct) as well as overall mean, and the minimum (min) and maximum (max) scores observed

Scene type	Mean	Min	Max
Landscapes	75.7	57.9	90
Fractals	55.7	42.1	80
Total	65.8	56.4	79.5

## Gaze analysis

The analysis of the gaze data consists of three parts. In the first part, basic fixation data are analyzed. In the second part, the fixation distributions are presented, which differ considerably from previous results obtained with images presented on a screen. In the third part, saccade patterns are analyzed to permit a direct comparison with the results of Foulsham and Kingstone (2010).

### Gaze measurements

Head and gaze data were recorded for 18 participants, for 160 trials per participants and 10 seconds viewing time per trial. None of the participants and none of the trials were excluded from the data analysis. The SMI gaze recording system suppresses values during intervals with missing data, for example, when the viewer is incorrectly positioned or during a blink. An analysis of the raw data showed that approximately 7.5% of data were suppressed, and in 6.8% of all trials of all participants, 20% or more of the data points were missing. Given our focus on spatial rather than temporal accuracy, this rate of missing data was deemed acceptable, especially in light of the fact that there were able to analyze approximately 6.6 million gaze measurements.

### Basic fixation data

An analysis of the number of fixations showed that participants made on average 3.75 fixations per second for the landscape scenes and 3.23 fixations per second for the fractal scenes, *F*(1,17) = 20.0, *p* < 0.001, *η^2^ =* 0.212. There was no effect of scene rotation, *F*(7,17) = 0.48, *p* = 0.851. Fixation duration was on average 199 ms for the landscape scenes and 235 ms for the fractal scenes, *F*(1,17) = 11.1, *p* = 0.004, *η^2^* = 0.159. There was a weak but significant effect of scene rotation, *F*(7,17) = 2.90, *p* = 0.008, *η^2^* = 0.012, with fixation durations for scene orientations 0° and 180° being somewhat longer than for the other orientations. In summary, participants made fewer and longer fixations for the fractal scenes than for the landscape scenes.

### Fixation distribution

**Figure 4.**
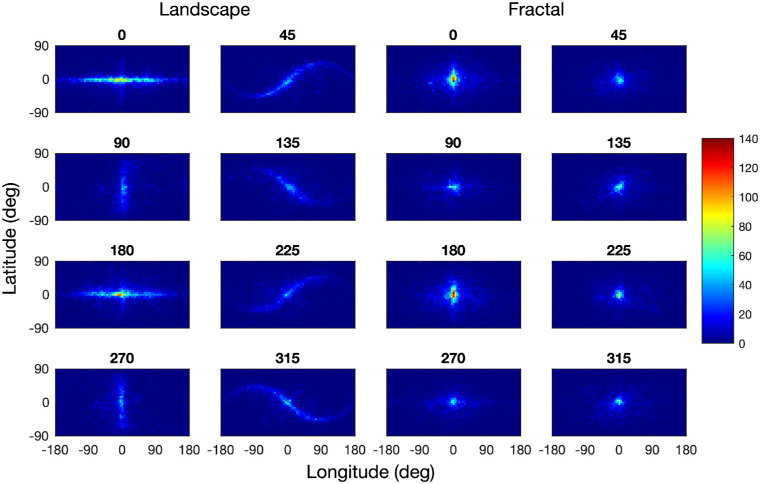
Distribution of fixations for landscape images (left) and fractal images (right), for all scene rotations in the range 0° to 315°, using a bin size of 5° longitude by 5° latitude. Frequencies have been normalized across all conditions, with dark blue pixels corresponding to zero fixation counts and dark red corresponding to a bin count of more than 140. The fixation distributions are best compared with the images in Figure 3.

The distribution of fixations differed substantially between the landscape and fractal scenes (Figure 4). The panels on the left show the bivariate fixation distributions for the landscape scenes for the eight scene orientations, those on the right for the fractal scenes. The distribution patterns for the landscape scenes show that participants tended to fixate along the horizon of these images. The fixation distributions for scene rotations 45°, 135°, 225°, and 315° are best understood by comparing them with the rotated panorama maps in Figure 3. The fixation distributions for scene rotations 90° and 270° are concentrated along the 0° meridian with little spill over to the 180° meridian, which is plausible given the physical constraints on the neck extension and flexion. A principal component analysis shows that the orientations of the fixation distributions (as defined by the angle to the first eigenvector) are all within a range of ±3.5° of the scene rotation, confirming the close match between the orientation of the fixation distributions and scene rotation. The fixations patterns for the landscape images are closely related to those obtained by Sitzmann et al. (2018), who found a similar horizon bias. In contrast with the landscape scenes, the fixation patterns for the fractal scenes are much closer to isotropic, and there is no evidence of the fixation patterns being aligned with the virtual horizon or a symmetry axis of the panoramas.

As shown in Table 2, the spread of fixations along the scene horizons (longitude) was much larger than the spread in the orthogonal direction (latitude): For landscapes, the ratio of *SD* Longitude to *SD* Latitude of approximately 3.0, whereas for fractal scenes, the ratio was approximately 1.6, and thus decreased as compared with the landscape scenes.

**Table 2.** Standard deviations of the fixation distributions, in longitude and latitude for the two scene types. The differences between *SD* longitude and *SD* latitude are statistically significant for both scene types, all *p* < 0.001

Scene type	*SD* Longitude (deg)	*SD* Latitude (deg)	*t*(17)	Cohen's *d*
Landscapes	64.82	21.76	11.83	3.55
Fractals	47.10	29.59	10.22	1.14

### Saccade direction

**Figure 5.**
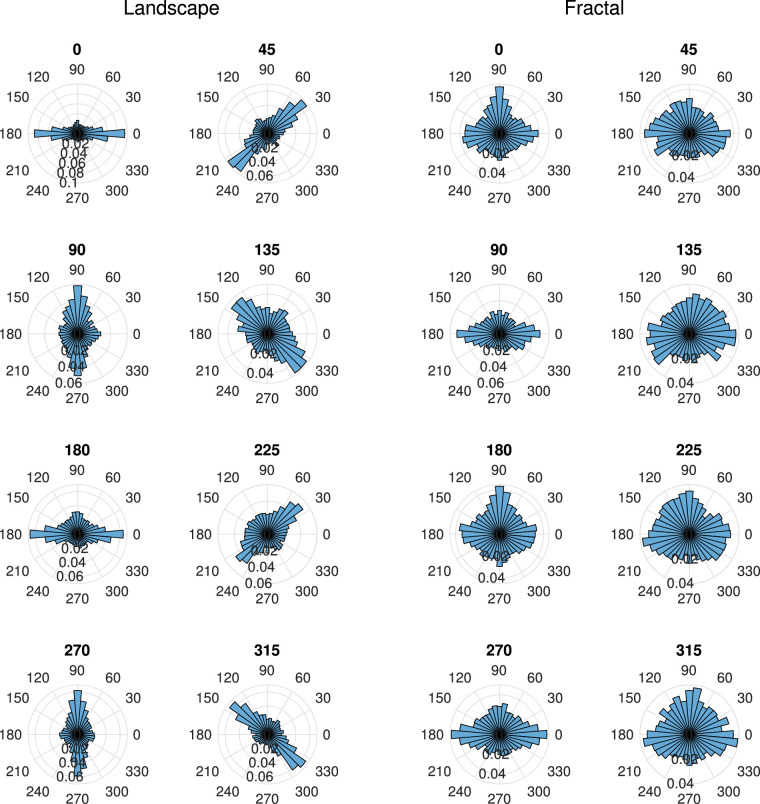
Polar histogram of saccade directions in world coordinates, for the landscape scenes (left), and for the fractal scenes (right), for all scene rotations. The histograms indicate relative probabilities and use a bin size of 10°, and the spacing of the inner rings corresponding a probability difference of 0.02.

The saccade patterns are closely related to the fixation distributions, but permit a direct comparison with the results of Foulsham and Kingstone (2010). Given the large spread of fixations along the horizon of the landscape images, it is plausible that saccade directions also align with the scene horizons. This finding is illustrated in Figure 5, which shows polar histograms of saccade directions in world coordinates, for landscape scenes on the left, for fractal scenes on the right, and for all scene rotations. The histograms for the landscape scenes show that most saccades were made along the horizon direction of the panoramas (i.e., left and right for the scene rotation of 0°), thus, showing a strong effect of scene rotation. The histograms for the fractal scenes, however, show a very different effect of scene rotation. Saccades were made in all directions, with a slight preference for saccades along the cardinal directions in world coordinates, namely to the left, the right, and up. This dovetails with past work (Foulsham & Kingstone, 2010; Foulsham et al., 2008), and as the fractal images are isotropic on average, with no clear texture orientation along the horizon direction, any response biases likely reflect preferential scanning biases that are structural (i.e., oculomotor) and/or learned.

**Figure 6.**
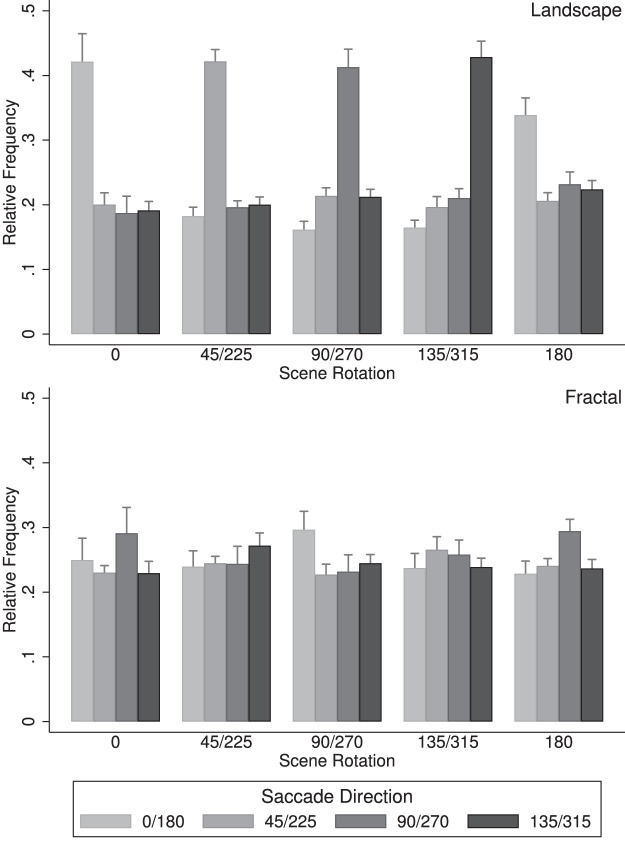
Saccade directions organized into four symmetric axes in world coordinates, for landscape scenes (top) and fractal scenes (bottom). Data show the mean proportions of saccades and standard error bars. Data show the mean proportions and standard errors of head shifts along the four saccade axes (0°/180°, 45°/225°, 90°/270°, and 135°/315°) and the five scene rotation groups (0°, 45°/225°, 90°/270°, 135°/315°, and 180°).

For the statistical analysis of the saccade directions, opposite saccade directions (0° and 180°, 45° and 225°, 90° and 270°, and 135° and 315°) and scene rotations (45° and 225°, 90° and 270°, and 135° and 315°) were combined to allow a direct comparison with the results of Foulsham et al. (2008) and Foulsham and Kingstone (2010) (see Figure 6). An overall analysis of variance yielded two significant effects, namely the interaction of scene rotation × saccade direction, *F*(12,204) = 64.12, *p* < 0.001, *η^2^* = 0.316, and the interaction of scene type × scene rotation × saccade direction, *F*(12,204) = 110.89, *p* < 0.001, *η^2^* = 0.408. A more detailed analysis revealed that for landscape scenes (Figure 6, left), there was a strong interaction of scene rotation with saccade direction, *F*(12,204) = 134.5, *p* < 0.001, *η^2^* = 0.851, with the saccade frequencies along the landscape horizons being approximately twice as high as those in the other directions. For the fractal landscapes (Figure 6, right), there was also a strong interaction of scene rotation with saccade direction, *F*(12,204) = 9.43, *p* < 0.001, *η^2^* = 0.174, with more frequent saccades in the cardinal directions for scene rotations 0°, 90°, 180°, and 270° and more isotropic saccade patterns for the other scene rotations.

The present study revealed strong patterns of saccade directions: For all rotations of the landscape scenes, saccades directions were primarily aligned with the scene horizons (Figure 5, left, and Figure 6, left), with 40.5% of the saccade directions within a range of ±22.5° of the horizon direction. For fractal scenes, saccades were more frequent in the horizontal and upward direction (in world coordinates) for scene rotations 0°, 90°, 180°, and 270°, an effect that was less pronounced for the other scene rotations. The saccade directions were, however, not aligned with the scene horizon, as was the case for the landscape scenes.

The results obtained for the landscape scenes in the present study are similar to those obtained by Foulsham et al. (2008), who found the saccade directions tended to be aligned with the horizon. For our fractal scenes, which show biases along cardinal coordinates, the results are convergent with past works (for instance, see Foulsham & Kingstone, 2010, Figures 2 and 3).

### Saccade amplitudes

The fixation distributions for landscape images shown in Figure 4 suggest (a) that saccades in the direction of the horizon are more frequent, as discussed in the previous section, and (b) that saccade amplitudes may also be larger in that direction. In contrast, the saccade directions and amplitudes may be more balanced for fractal landscapes.

**Figure 7.**
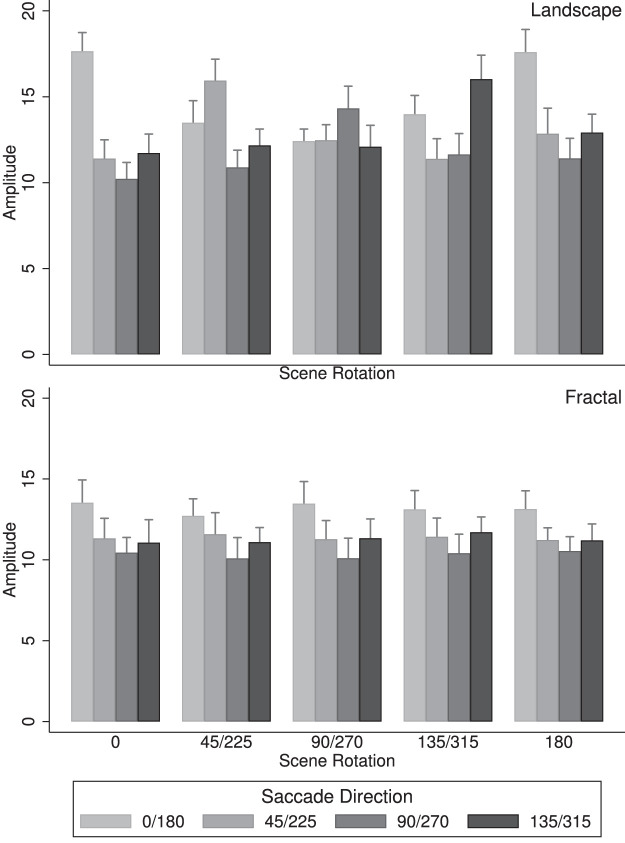
Saccade amplitudes along four axes in world coordinates, for landscape scenes (top) and fractal scenes (bottom). Data show the mean amplitudes of saccades (in degrees) and standard error bars along each axis and for the five scene rotation groups across participants.

Figure 7 illustrates the saccade amplitudes and is organized in the same way as the results for the saccade directions, with saccade amplitudes for landscape scenes shown in (Figure 7, left) and saccade amplitudes for fractal scenes shown in (Figure 7, right). An overall analysis of variance of saccade amplitudes yielded two significant main effects, a main effect of scene type, *F*(1,17) = 26.85, *p* < 0.001, *η^2^* = 0.074, with saccade amplitudes for landscape panoramas, *M* = 13.13, being larger than for fractal panoramas, *M* = 11.54; and a main effect of saccade direction, *F*(3,51) = 57.03, *p* < 0.001, *η^2^* = 0.147, indicating that saccade amplitudes depended on saccade direction. There were also two significant interactions, namely Scene rotation × Saccade direction, *F*(12,204) = 26.23, *p* < 0.001, *η^2^* = 0.093, indicating that saccade amplitudes depended on scene rotation and saccade direction, and Scene type × Scene rotation × Scene direction, *F*(12,204) = 25.27, *p* < 0.001, *η^2^* = 0.078, indicating that these effects depended on scene type. These results are best understood through separate analyses of the landscape and the fractal scenes. An analysis of the landscape scenes (Figure 7, left) revealed a main effect of saccade direction, *F*(3,51) = 64.6, *p* < 0.001, *η^2^* = 0.151, indicating that saccade amplitudes changed with saccade direction, and a significant interaction of scene rotation with saccade direction, *F*(12,204) = 37.5, *p* < 0.001, *η^2^* = 0.302, with the saccade amplitudes along the landscape horizons larger than those in the other directions. For the fractal landscapes (Figure 7, right), there was also a significant main effect of saccade direction, *F*(3,51) = 25.38, *p* < 0.001, *η^2^* = 0.173, with higher saccade amplitudes in the horizontal direction (in world coordinates).

For landscape scenes, saccade amplitudes were on average 13.1° and fixation durations were 199.1 ms; for fractal scenes, saccade amplitude were on average 10.8° and fixation durations were 234.9 ms. The inverse relationship between saccade amplitudes and fixation durations has been reported before (e.g., Unema, Pannasch, Joos, & Velichkovsky, 2005; Tatler & Vincent, 2008). It suggests that global scanning—characterized by large amplitude saccades and short fixation durations—was prevalent for the landscape scenes, while more local scanning—characterized by smaller amplitude saccades and longer fixation durations—was prevalent for the fractal scenes.

### Interindividual differences of gaze distributions

**Figure 8.**
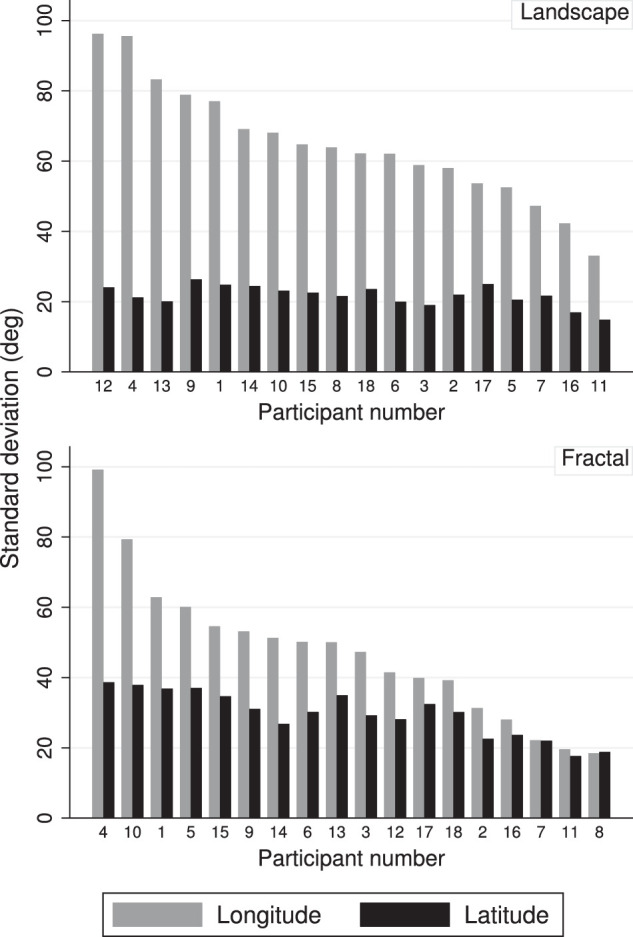
Distribution of gaze spread by participant, for landscapes (top) and fractal scenes (bottom). The graphs show the standard deviations of latitude and longitude for the two scene types, separately for each participant. The bars are ordered by the size of the longitude standard deviation.

Participants differed with respect to the spread of their fixation patterns in the sense that some participants explored a wide area of the scenes, while others explored only a small fraction. This is illustrated in Figure 8, which shows the standard deviations of the longitudes and latitudes of the fixation patterns, separately for the two scene types and for all participants. The bars are ordered by the size of the longitude standard deviations. The differences between participants are very large for the longitudes (by a factor of about 5 for the fractal scenes, and a factor of about 3 for the landscape scenes), and smaller for the latitudes (by a factor of about 2 for the fractal scenes and a factor of about 1.5 for the landscape scenes).

### Summary

The results of the gaze analysis show that the fixations and saccades are determined by the content of the panoramas: In landscape panoramas, the fixation distribution and the most frequent saccade direction are aligned with the horizon, even when the panoramas are rotated about the view axis. The alignment is precise, that is, within at most a few degrees of the rotation angle. In fractal panoramas, which lack an identifiable horizon, the fixation distributions are somewhat closer to isotropic, with a tendency of saccade directions along the cardinal directions, and the largest amplitudes to be horizontal, in world coordinates. There was considerable variation among participants in terms of scene exploration.

## Head analysis

**Figure 9.**
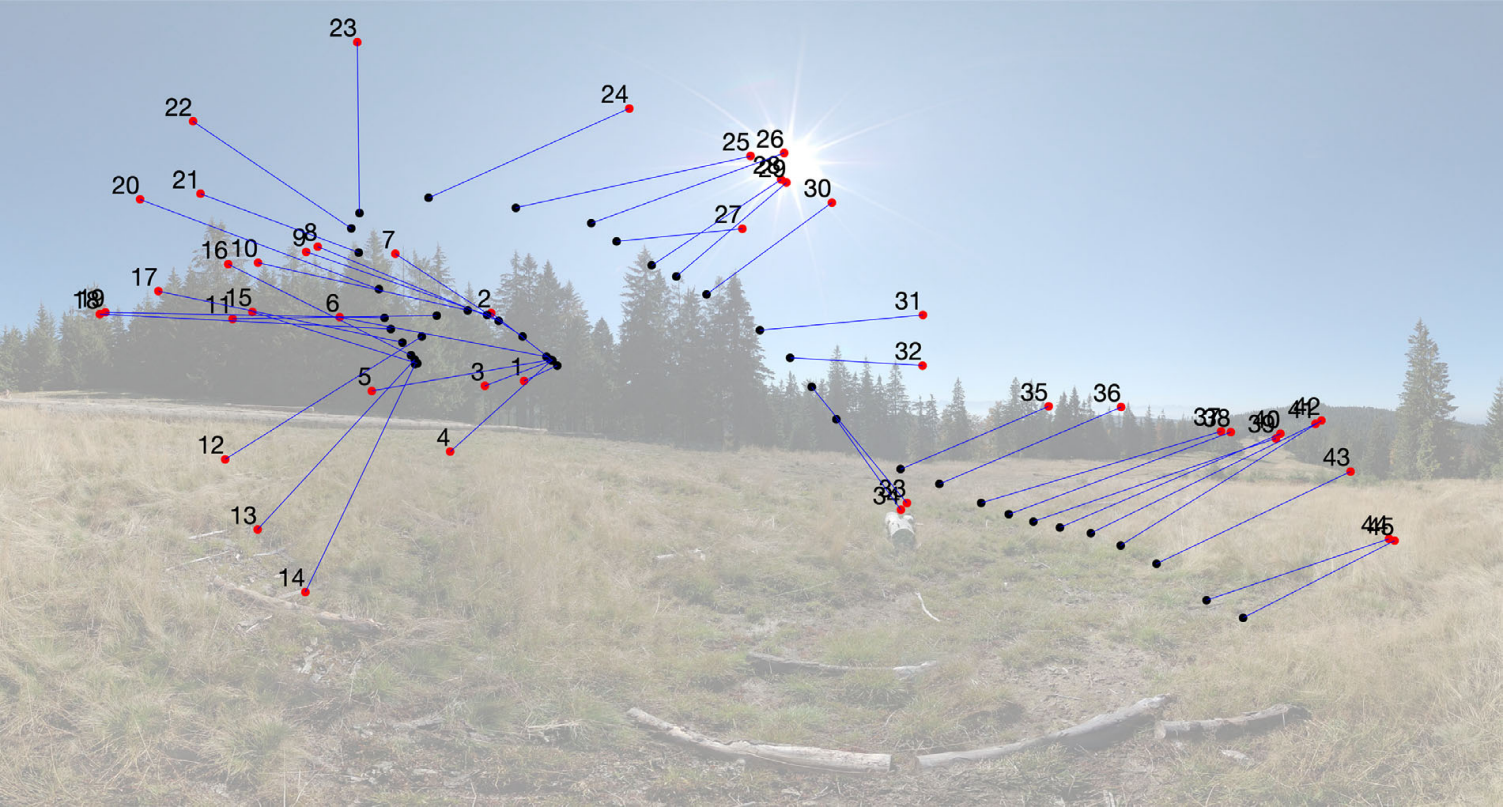
Example of (gaze) fixations and the corresponding head fixations. The red circles show a sequence of fixations (numbered from 1 to 45). The black circles show the head fixations during each fixation, and the lines connect the two.

As described elsewhere in this article, the location and orientation of the VR headset were measured using two infrared base stations situated in opposite corners of the experiment room. From the location and orientation of the headset, one can determine where on the stimulus sphere the head is pointing. This point is referred to as head hit point or head point. Head movements are not ballistic in the way that eye movements are. For this reason, there are, in contrast with gaze, no natural demarcations for head shifts and head fixations. To compare head with gaze, we adopted the convention that, for every gaze fixation, we define head fixation as the average head point during that fixation. Eye fixations and head fixations for one participant and a landscape stimulus are shown in Figure 9. The red circles show a sequence of eye fixations (numbered from 1 to 45), the black circles show the head fixations during those fixations, and the lines connect fixations with the corresponding head fixations. The lines thus represent differences between gaze and head, and are determined by the eye direction within the headset.

### Distribution of head fixations

**Figure 10.**
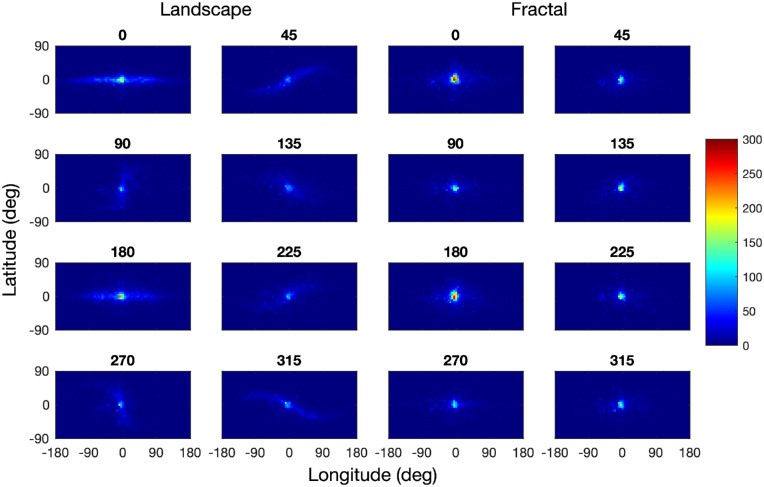
Distribution of head fixations for landscape images (left) and fractal images (right), for all scene rotations in the range 0° to 315°, using a bin size of 5° longitude by 5° latitude. Frequencies have been normalized across all conditions, with dark blue pixels corresponding to zero fixation counts and dark red corresponding to bin count of more than 300. The distributions are best compared with the images in Figure 3.

The distribution of head fixations differed substantially between the landscape and fractal scenes (Figure 10). The panels on the left show the bivariate distributions of head positions for the landscape scenes for the eight scene orientations, those on the right for the fractal scenes. The distribution patterns for the landscape scenes show that participants tended to fixate along the horizon of these images. The distributions of head positions for scene rotations of 45°, 135°, 225°, and 315° are best understood by comparing them to the rotated panorama maps in Figure 3.

An informal comparison of Figure 10 with Figure 4 indicates that the range of eye fixations is larger than the range of head fixations. The statistical analysis in Table 3 shows that the standard deviations of the eye fixations are significantly larger than those of the head fixations, for both scene types, and for longitudes and latitudes. For ease of comparison, the gaze spreads reported in Table 1 are repeated in Table 3. The smaller range of head movements suggests that they are used to center the visual field on a particular point in the environment, and eye movements are used explore the visual field. This results in the range of fixations exceeding the range of head positions. Apart from these quantitative differences, the distributions of eye fixations and head positions are, however, qualitatively similar.

**Table 3.** Fixation and head fixation spreads in longitude and latitude for the two scene types. All differences between *SD* Gaze and *SD* Head are statistically significant (all *p* < 0.001), for both scene types and both, longitudes and latitudes

Scene type	Longitude/Latitude	*SD* Gaze	*SD* Head	*t*(17)	Cohen's *d*
Landscape	Longitude	64.82	54.54	13.93	0.585
Landscape	Latitude	21.76	19.51	4.47	0.659
Fractal	Longitude	47.10	38.36	10.22	0.426
Fractal	Latitude	29.59	24.30	8.38	0.739

### Direction of head shifts

**Figure 11.**
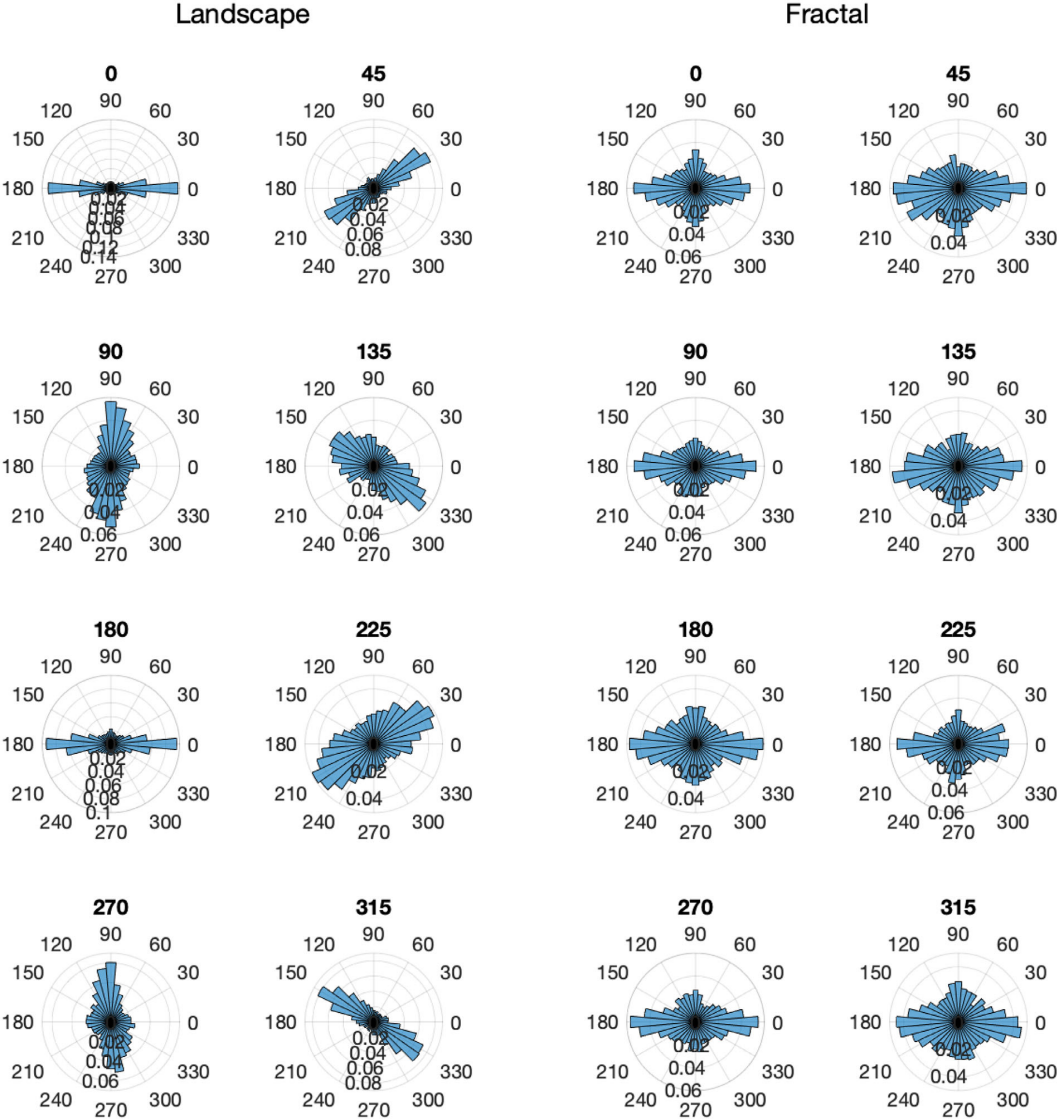
Direction distribution of head shifts in world coordinates, for the landscape scenes (left), and for the fractal scenes (right), for all scene rotations. The histograms indicate relative probabilities and use a bin size of 10°, and the spacing of the inner rings corresponding a probability difference of 0.02.

In analogy to the gaze analysis, we now describe the head shift patterns, allowing again a direct comparison with results obtained in previous studies. Figure 11 shows polar histograms of head shift directions, for landscape scenes on the left, for fractal scenes on the right, and for all scene rotations. The histograms for the landscape scenes show that most movements were made along the horizon of the images (i.e., left and right for the scene rotation of 0°), thus showing a strong effect of scene rotation. The histograms for the fractal scenes show no systematic effect of scene rotation. Head shifts were made in all directions, with a slight preference for the cardinal directions in world coordinates, that is, they are determined by the gravitational vertical (Cristino & Baddeley, 2009). This pattern of results can be attributed to the fact that the fractal images were isotropic on average, with no clear horizon-defined anisotropy. These results for head shifts are similar to those of the saccades. It is interesting to speculate that shared similarities for the fractal images, which are not unique to the head or eyes, point toward scanning patterns that are learned rather than structural (e.g., mechanisms that move the head or eyes).

**Figure 12.**
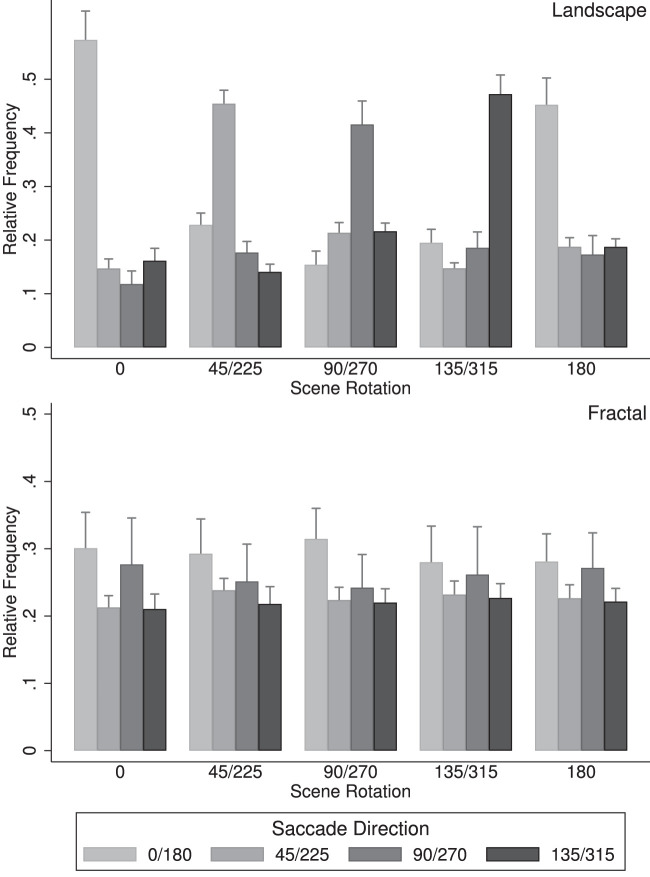
Head shift directions organized into four symmetric axes in world coordinates, for landscape scenes (top) and fractal scenes (bottom). Data show the mean proportions and standard errors of head shifts along the four head shift axes (0°/180°, 45°/225°, 90°/270°, and 135°/315°) and the five scene rotation groups (0°, 45°/225°, 90°/270°, 135°/315°, and 180°).

For the statistical analysis of head shifts, head shift in opposite directions (0° and 180°, 45° and 225°, 90° and 270°, and 135° and 315°) and scene rotations (45° and 225°, 90° and 270°, and 135° and 315) were combined to allow a direct comparison with results obtained previously (Foulsham et al., 2008; Foulsham & Kingstone, 2010). Figure 12 shows the relative frequencies along the four head shift axes (0°/180°, 45°/225°, 90°/270°, and 135°/315°) and the five scene rotation groups (0°, 45°/225°, 90°/270°, 135°/315°, and 180°), for landscape scenes in (Figure 12, left) and the fractal scenes (Figure 12, right).

An overall analysis of variance yielded three significant effects, namely the effect of head shift direction, *F*(3,51) = 6.96, *p* < 0.001, *η^2^* = 0.077, indicating that head shifts occurred with different frequencies along the four axes. The interaction Scene rotation × Head shift direction, *F*(12,204) = 88.67, *p* < 0.001, *η^2^* = 0.284, and the interaction Scene type × Scene rotation × Head shift direction, *F*(12,204) = 106.07, *p* < 0.001, *η^2^* = 0.288, were also significant and are best understood by analyzing landscape scenes and fractal scenes separately. For landscape scenes (Figure 12, left), there was a significant effect of head shift direction, *F*(3,51) = 22.01, *p* < 0.001, *η^2^* = 0.081, and a significant interaction of Scene rotation × Head shift direction, *F*(12,204) = 153.2, *p* < 0.001, *η^2^* = 0.770, both resulting from the fact that head shifts along the landscape horizons were approximately three times as frequent as those in the other directions. For the fractal landscapes (Figure 12, right), there was a marginal effect of head shift direction, *F*(3,51) = 2.73, *p* = 0.054, *η^2^* = 0.116, with head shift directions in the cardinal directions (0°, 90°, 180°, and 270°, in world coordinates) being somewhat higher than in the other directions.

For landscape scenes, the frequencies of head shifts along the horizons were more frequent than for the other directions, and there was no systematic difference between the other three directions. This effect is the same as found for the saccade directions (Figure 6), but even more biased toward movements along the horizon line: 47.9% of the head shifts were within ±22.5° of the horizon line, but only 42.1% of the saccades, *t*(142) = 4.204, Cohen's *d* = 0.701. For the fractal scenes, there was a stronger bias toward movements in the cardinal directions (horizontal and vertical) in world coordinates than was the case for saccades.

### Amplitude of head shifts

**Figure 13.**
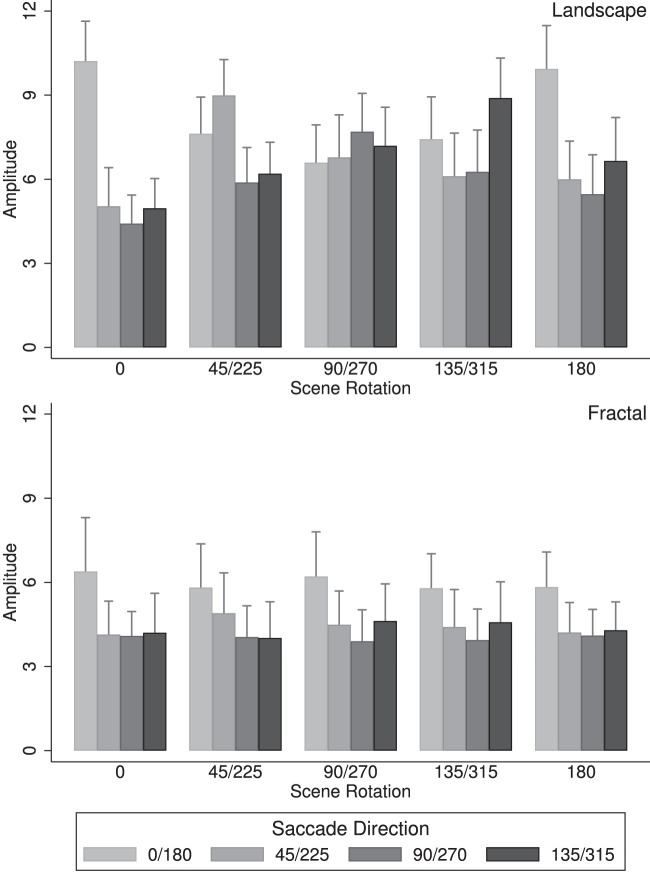
Amplitudes of head shifts along four axes in world coordinates, for landscape scenes (top) and fractal scenes (bottom). Data show the mean amplitudes of head shifts and standard error bars along the four head shift axes (0°/180°, 45°/225°, 90°/270°, and 135°/315°) and the five scene rotation groups (0°, 45°/225°, 90°/270°, 135°/315°, and 180°).

Head shifts were defined as the differences between successive head fixations. Figure 13 illustrates the head shift amplitudes along the four head shift axes (0°/180°, 45°/225°, 90°/270°, and 135°/315°) and the five scene rotation groups (0°, 45°/225°, 90°/270°, 135°/315°, 180°), for landscape scenes in (Figure 13, left) and the fractal scenes (Figure 13, right). An overall analysis of head shift amplitudes yielded several significant effects, namely an effect of scene type, *F*(1,17) = 34.3, *p* < 0.001, *η^2^ =* 0.128, with an average head shift amplitudes *M* = 6.92° for the landscape scenes and *M* = 4.70° for the fractal scenes, that is, head shift amplitudes were on average large for landscape scenes than for fractal scenes. Two interactions were significant, namely Scene rotation × Head shift direction, *F*(12,204) = 25.45, *p* < 0.001, *η^2^ =* 0.048, and Scene type × Scene rotation × Head shift direction, *F*(12,204) = 18.28, *p* < 0.001, *η^2^ =* 0.033. These interactions are best understood by analyzing the results for landscape scenes and fractal scenes separately. An analysis of the landscape scenes revealed an effect of scene rotation, *F*(4,68) = 3.89, *p* = 0.007, *η^2^ =* 0.015; a significant effect of head shift direction, *F*(3,51) = 83.04, *p* < 0.001, *η^2^ =* 0.082; and a significant interaction Scene rotation × Head shift direction, *F*(12,204) = 32.59, *p* < 0.001, *η^2^ =* 0.157. These results are in part owing to the fact that the amplitude of head shifts along the scene horizons were larger than in the other directions. An analysis of the fractal scenes revealed a significant effect of head shift direction, *F*(3,51) = 33.11, *p* < 0.001, *η^2^ =* 0.084; and a weak effect of the interaction Scene rotation × Head shift direction, *F*(12,204) = 1.78, *p* = 0.054, *η^2^ =* 0.006. These results indicate that head shift amplitudes along the horizontal direction (in world coordinates) are larger than in the other direction, and, in contrast with the landscape scenes, there was no effect of scene rotation on head shift amplitudes. Taken together, the results show that, for landscape scenes, the amplitude of the head shifts were greatest for saccades in the direction of the horizon while for fractal scenes, the amplitudes were largest for head shifts in the horizontal direction (in world coordinates).

### Interindividual differences of head fixations

**Figure 14.**
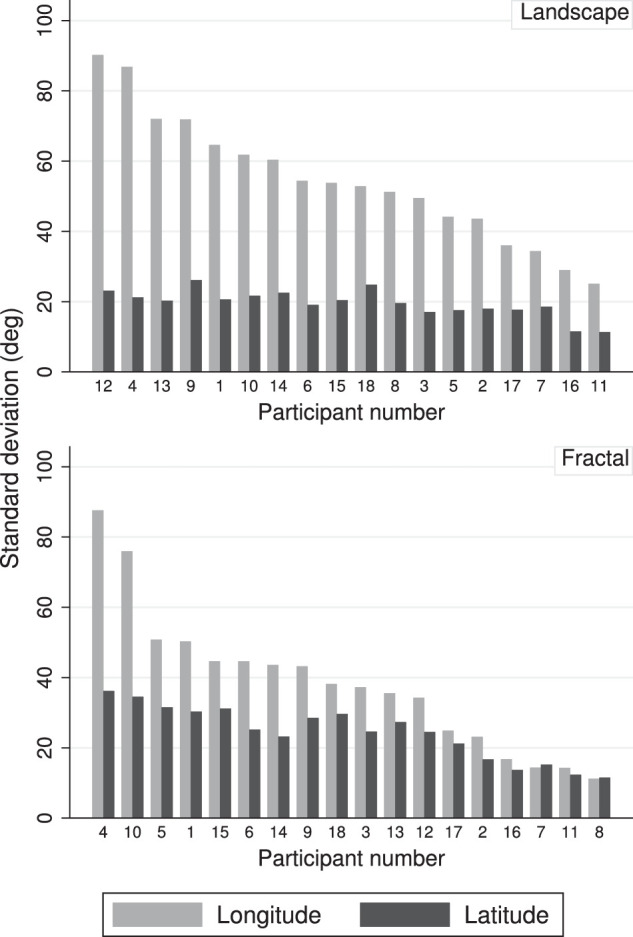
Distribution of head spread by participant, for landscapes (top) and fractal scenes (bottom). The graphs show the standard deviations of latitude and longitude for the two scene types, separately for each participant. The bars are ordered by the size of the longitude standard deviation.

Participants differed with respect to their head movement patterns in the sense that some participants moved their head a lot during the exploration of the panoramas, while others kept their head fairly still while they explored the panoramic scenes. This is illustrated in Figure 14, which shows the standard deviations of the longitudes and latitudes of the head distributions, separately for the two scene types and for all participants. The bars are ordered by the size of the longitude standard deviations. The differences between participants are very large for the longitudes (by a factor of about 8 for the fractal scenes, and a factor of about 4 for the landscape scenes), but much smaller for the latitudes (by a factor of about 3 for the fractal scenes and a factor of about 2 for the landscape scenes).

### Head roll

We observed that some participants somewhat tilted their head (i.e., rolled their head), consistent with an attempt to see the scenes closer to their canonical (upright) orientation. For example, if a scene is rotated by 45° then, ignoring ocular torsional eye movements, a head rotation of 45° would result in an upright orientation of the scene within the visual field. If this observation is correct then the head rolls should occur in the direction of the scene rotations. This adjustment should happen gradually during the stimulus presentation.

**Figure 15.**
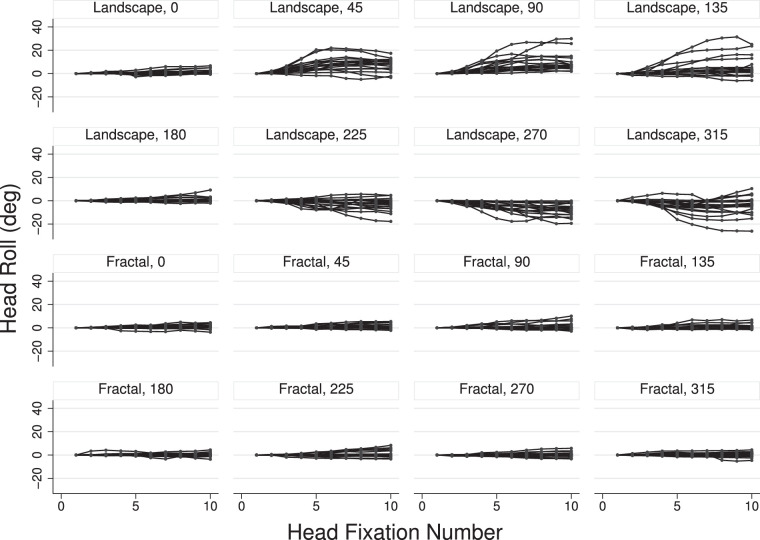
Head rolls as a function of head fixation number. The graphs show individual head roll curves that are normalized relative to the head roll for the first head fixation, with each curve showing head rolls for one participant. The responses for landscape scenes are shown in the top half and for fractal scenes in the bottom half.

Figure 15 shows individual curves of head rolls as a function of head fixation number and relative to the head rotation for the first fixation. The effect is quite strong for the landscape scenes and virtually nonexistent for the fractal scenes. For scene orientations 45° and 90°, the head was on average rotated up to about 10° counterclockwise, and for scene orientations 270° and 315°, the head was rotated on average up to about 7° clockwise. These findings converge with the notion that when head rotation occurs, it is performed to bring the scenes closer to the upright orientation.

To examine the rate of head roll, the first 10 head fixations of the rotation data were analyzed using a mixed-effects linear regression analysis, which yielded regression slopes significantly different from zero and a magnitude exceeding 0.4°/fixation for the landscape scenes and for scene rotations 45°, 90°, 135°, 225°, 270°, and 315°. For the other scene rotations of the landscape scenes, and for the fractal scenes, the slopes were either nonsignificant or smaller than 0.4° per fixation.

### Summary

Head fixations and movements are determined by the content of the panorama, in a manner that is remarkably similar to what was observed for the gaze data. In landscape panoramas, head fixations, direction, and movement amplitude show a pronounced tendency to be in alignment with the horizon, even when the panoramas are rotated around the z-axis of the virtual space. This was complemented by the tendency of observers to rotate their heads to bring the landscape horizons somewhat closer to the canonical upright orientation. This pattern of fixations, movements, and head rotations was absent for the fractal images, which lack a well-defined horizon line. Instead, the direction of head movements tended to be in the cardinal directions, and the amplitude of the head movements tended to be in the horizontal direction, in world coordinates.

## Gaze and head

The previous sections analyzed head fixation patterns and eye fixation patterns separately, and as noted, they are remarkably similar. In this section, the focus is on the relation between head and gaze. One example of the relation between head and gaze is shown in Figure 9. Several observations are noteworthy: The head positions change only slowly; the head positions vary in a smaller range than the gaze positions (see also Table 3); the head and gaze fixations coincide only in rare cases; and finally, the head fixations seem to lag behind the gaze fixations. These observations are explored more formally in this section.

### Gaze–head: Distance analysis

**Figure 16.**
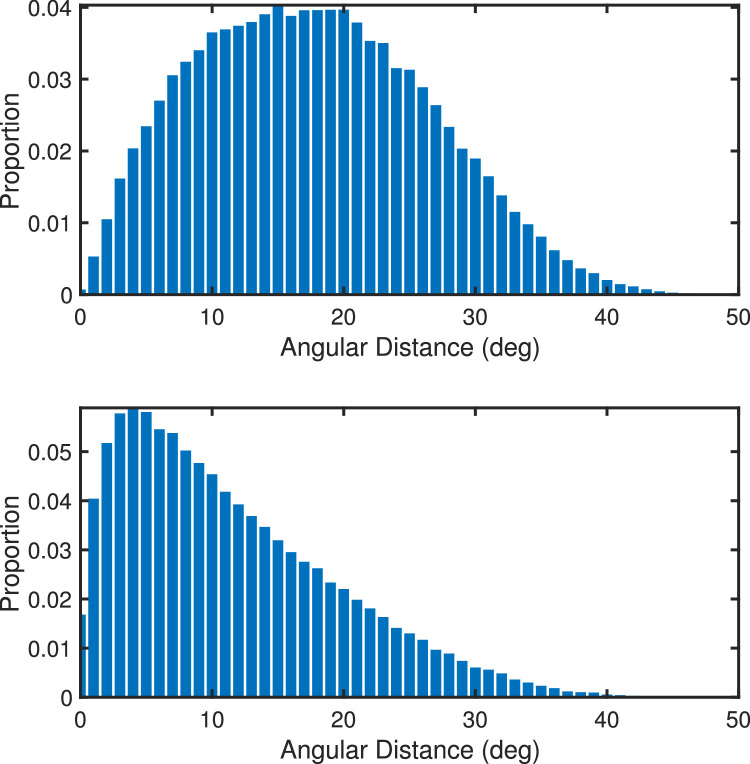
Top: Histogram of all gaze-head distances that correspond in time. Bottom: Histogram of the minimum gaze-head distances within a window of ±8 fixations. The angular distances in both panels have been averaged across scene types, scene rotations, and participants.

Head movements are used to reposition the visual field in different directions of the environment and eye movements serve to explore the visual field defined by the head position. It is thus interesting to explore the range of fixations around the center of the visual field when the head is allowed to move freely. The top of Figure 16 shows a histogram of all angular gaze-head fixation distances averaged over both scene type, all eight scene rotations, and all participants. To be clear, the histogram is based on the distances illustrated in Figure 9, that is, the distances between head positions and gaze positions that correspond in time. The angular distances are computed as great-circle distances between gaze positions and head positions (Appendix A). The results show that gaze and head positions coincide only rarely, and the most frequent head-gaze distances are found to be in the range of about 10° to 20°, *Mode* = 15, *Md* = 17.3.

The comparison between head and gaze positions at the same time point may, however, be somewhat misleading, as will be shown below, the head tends to lag behind gaze. Thus it may be more appropriate to analyze the minimum angular distances between gaze and head positions within a time window of approximately 2 seconds (i.e., ±8 fixations, Appendix B). When examined in this way, one finds that the distances between head and gaze positions are dramatically reduced, with the most frequent minimum distances in the range 2° to 6°, *Mode* = 4, *Md* = 8.7 (as illustrated in the lower panel of Figure 16). In sum, these results show that the angular distance between gaze and head is fairly small, with a mode of 4°, indicating that a fairly local range of locations close to the center of the head-defined visual field is explored. Both panels in Figure 16 are based on approximately the same number of data points (about 100,000).

### Gaze–head: Lag analysis

**Figure 17.**
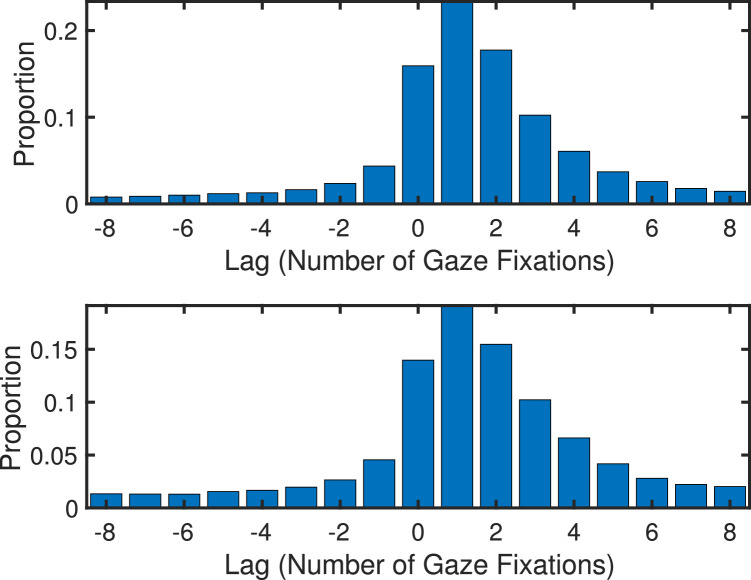
Histogram of gaze-head lags for landscape scenes (top) and fractal scenes (bottom), with the lag expressed in number of gaze fixations. If gaze is closer to a later head position, that is, has a positive lag, then gaze is leading. If gaze is closer to an earlier head position, that is, has a negative lag, then gaze is trailing. The results show that gaze is leading head in most cases, with a peak lag of about one to two fixations, corresponding to about 200 ms.

To determine whether, and by how much, gaze was leading or following the head, gaze and head fixations were compared using a minimum distance analysis: Given a (gaze) fixation *g_i_* and a set of head fixations *h_j_* before and after the time point *i*, the $h_{j}^{min}$ with minimum distance was determined. If the $h_{j}^{min}$ occurs before *g_i_* then it is inferred that head is leading gaze, otherwise it was concluded that head is lagging behind gaze. The results of this minimum distance analysis for landscape images are shown in Figure 17, indicating that most minimum distances occurred at a positive lag, that is, the gaze positions were most often leading the head positions. The results also show that the lag peak is around one to two fixations, suggesting that the eye leads the head by approximately 200 ms. Further details of the minimum distance analysis are presented in Appendix B.

Previous work on the coordination between eye and head movements suggest that for relatively small eye movements (<45°) the eye leads the head, and for larger shifts (>60°) the initiation of the two tends to be more synchronous (Barnes, 1979). In both cases, however, the eyes terminate well in advance of the slower head movements owing to longer contraction times for the neck muscles and the greater inertial forces acting on the head compared with the eye (Bizzi et al., 1971; Gilchrist, Brown, Findlay, & Clarke, 1998; Freedman, 2008). Interestingly, the conditions that result in the head leading the eyes are relatively few. Such situations include: repetitive, predetermined events, such as watching a tennis match (Morasso, Sandini, Tagliasco, & Zaccaria. 1977; Pelz, Hayhoe, & Loeber, 2001); preparation for a specific task-oriented event, such as shoulder checking in a car before changing lanes (Doshi & Trivedi, 2012); or choosing to move the eyes into space that is outside visible range, such as when looking at the world through binoculars (Solman et al., 2017). None of these unique conditions exist within the present study, and indeed, as seen in Figure 16, our participants' eye movements tended to be <45°. Thus, our finding that gaze leads the head is convergent with a wealth of past research.

### Eye in head

**Figure 18.**
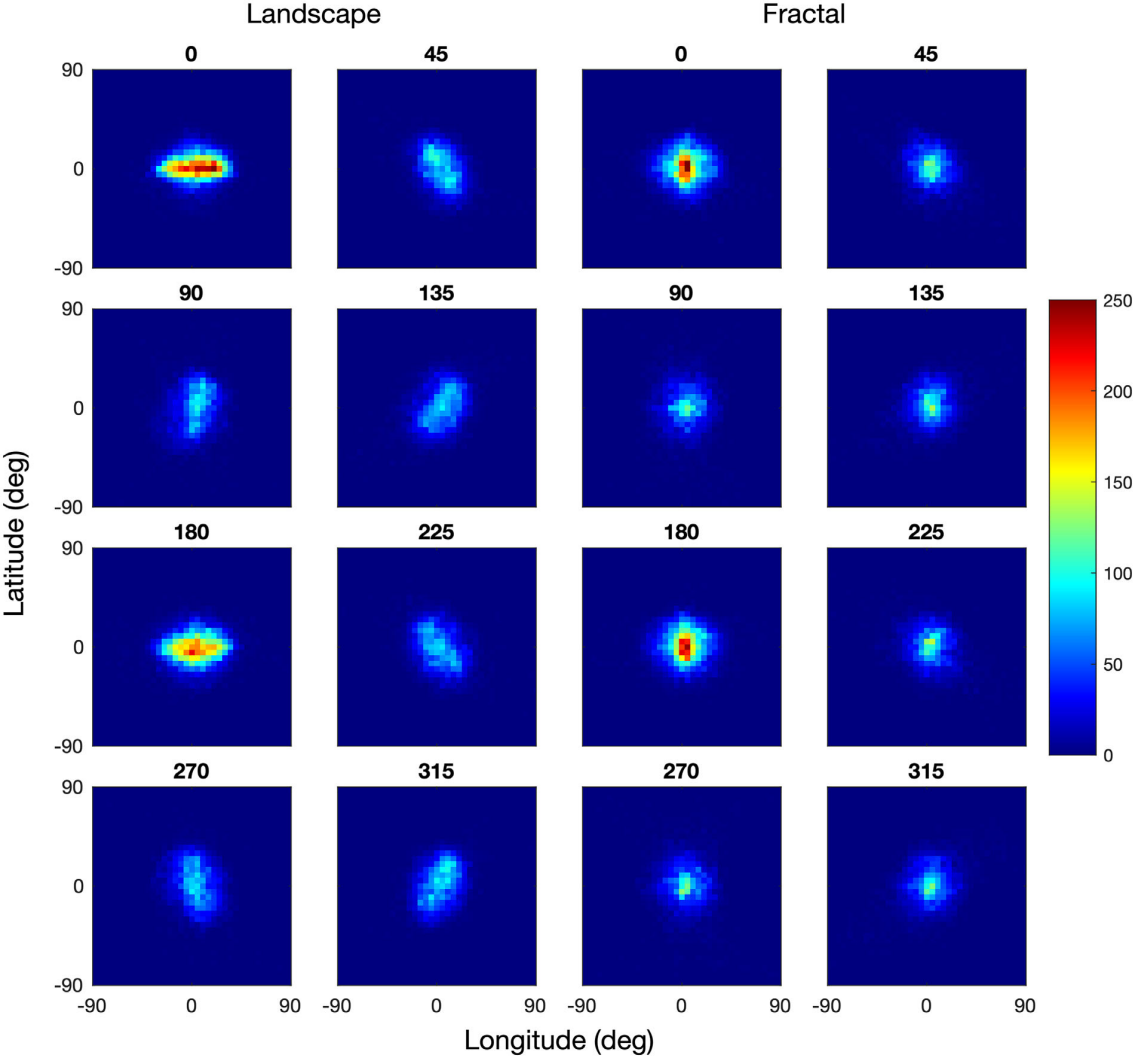
Distribution of fixations in head coordinates, for landscape images (left) and fractal images (right), for all scene rotations in the range 0° to 315°, using a bin size of 5° longitude by 5° latitude. Frequencies have been normalized across all conditions, with dark blue pixels corresponding to zero fixation counts and dark red corresponding to bin count of more than 250. The distributions are best compared with Figures 3, 4 and 10.

In the section on Gaze Analysis, the distributions of fixations were shown in world-coordinates, and in the section on Head Analysis, the distribution of head positions were also shown in world-coordinates. To further explore the relation between gaze and head, we now analyze the distribution of gaze in a head-centered coordinate system by calculating the angular difference between gaze points and head points. With this approach, we can express eye points in the same coordinate system as gaze points and head points. Figure 18 illustrates the distributions of fixations in head coordinates, which indicates that the fixation distribution differed substantially between the landscape and fractal scenes. The panels on the left show the bivariate fixation distributions for the landscape scenes for the eight scene orientations, those on the right for the fractal scenes. The fixation distributions are best understood by comparison with the rotated panorama maps in Figure 3, the distribution of fixations in world coordinates in Figure 4, and the distribution of head positions in Figure 10. The distribution patterns for the landscape scenes show that participants tended to fixate along the horizon of these panoramas, whereas they are much closer to isotropic for the fractal panoramas.

As shown in Table 4, the spread of eye positions along the scene horizons (longitude) was much larger than the spread in the orthogonal direction (latitude): For landscapes, the ratio of *SD* longitude to *SD* latitude of approximately 1.5, while for fractal scenes, the ratio was approximately 1.4, thus somewhat reduced compared with the landscape scenes.

**Table 4.** Standard deviations of the eye-in-head distributions, in longitude and latitude for the two scene types. The differences between *SD* Longitude and *SD* Latitude are statistically significant (all *p* < 0.001) for both scene types

Scene type	*SD* Longitude (deg)	*SD* Latitude (deg)	*t*(17)	Cohen's *d*
Landscapes	18.51	11.99	15.80	2.39
Fractals	18.51	13.26	5.12	1.22

### Summary

The previous sections had analyzed head fixation patterns and eye fixation patterns separately, and as noted, they largely mirror one another. In this section, the relation between head and gaze data was examined, and the results suggest a complementary relationship between head and eye. A temporal analysis of gaze and head based on minimum gaze-head distances suggests that, in the visual exploration of panoramic scenes, gaze is almost always leading the head. An analysis of the minimum distances between gaze and head suggests that gaze is used to explore a fairly local range within the head-defined visual field. The eye in head distributions revealed a general tendency for the eye to remain close to the center of the head, although this was sensitive to scene type and rotation. Finally, we were also able to show the typical VOR in our data.

## General discussion

We investigated visual exploration of omnidirectional panoramic scenes in a VR environment where the observers were allowed to freely move their head to inspect different areas of the panorama. We begin by discussing the different approaches to studying eye movement behavior and their advantages and disadvantages, followed by a characterization of head and gaze behavior in omnidirectional scenes, as well as its implications for mechanisms of visual exploration.

### Approaches to studying visual exploration

In the past, most studies on eye movement behavior have used static images or videos (e.g., Foulsham, Cheng, Tracy, Henrich, & Kingstone, 2010; Foulsham et al., 2011) presented on a screen. A majority of these studies measured eye movements while the observer's head was immobilized, usually by a chinrest. This study design has major implications for the study of eye movements. First, as Foulsham et al. (2011) have pointed out, gaze behavior without head movements may not reflect the dynamics of gaze selection in the real world, which involves head movements rather than large scanning eye movements (i.e., in real life people tend to look with their head and eyes, not with their eyes alone). Second, the visual information presented was preselected by the experimenter and was usually available in its entirety; hence, it did not require head movements to select new and unexplored areas of the visual environment. Third, the scanning behavior while passively watching static scenes shows very different characteristics compared with the scanning behavior while navigating through an environment. For example, eye movements were more centralized than for scenes presented on a monitor and were often centered on the heading point during walking (Foulsham et al., 2011; Foulsham & Kingstone, 2017).

When observers are allowed to freely move through their environments, looking behavior changes substantially from head-fixed passive viewing (e.g., walking through a university campus in Foulsham et al., 2011; see also ‘t Hart et al., 2009; ‘tHart & Einhäuser, 2012; Foulsham, Chapman, Nasiopoulos, & Kingstone, 2014). In this case, observers are faced with multiple tasks that need to be solved at the same time, namely, detecting and coding of objects nearby and far away, path planning using far objects, and obstacle avoidance of objects and people nearby. Although such studies are important, one major disadvantage is that, owing to physical and other constraints, the visual environment cannot be manipulated easily to test theories of visual exploration. For these reasons, it can be challenging to make inferences about the characteristics of visual exploration from real-world studies using mobile eye trackers.

The present article has focused on the visual exploration of omnidirectional panoramic scenes in a VR environment, where head movements were freely allowed to select areas of interest in the panoramas. By tracking the head and eyes in VR, we were able to analyze simultaneous head and eye movements and fixations, and study their coordination. Furthermore, the present design also allowed us to control the visual environment for experimental purposes, that is, to manipulate the content of the entire (360°) visual environment (landscapes and fractals) and to rotate these scenes around the view axis to determine their effects on viewing behavior.

### Characterization of gaze and head behavior in omnidirectional scenes

The analysis of gaze behavior revealed different fixation patterns for landscape scenes and fractal scenes: For landscape scenes, fixations were concentrated along the scene horizons, a finding that confirmed earlier results of Foulsham et al. (2011) and Sitzmann et al. (2018) for gaze distributions. For fractal scenes, however, the fixation distributions were close to being isotropic, a result that can be attributed to the statistically nonoriented nature of the fractal scenes used. A comparison of landscape and fractal scenes reveals that saccade amplitudes were larger and fixation durations shorter for the landscape scenes compared with the fractal scenes, suggesting that global scanning was more prevalent for landscape scenes, and local scanning more prevalent for fractal scenes (see Tatler & Vincent, 2008).

The analysis of head movements showed that the variance of the head fixations was smaller than for the (gaze) fixations, suggesting that the eyes were used to extend the range of gaze positions. An analysis of head shifts yielded results that paralleled those of gaze: For landscape scenes, most head shifts were made in the direction of the scene horizons, with head shift amplitudes being larger than in the other directions. For fractal scenes, head shifts were more likely in the cardinal directions and the largest amplitudes in the horizontal direction (in world coordinates). An analysis of head roll showed that the head tended to be rotated in counter-clockwise direction for scene rotations 45° to 135°, and clockwise for scene rotations 225° to 315°, in an attempt to bring the landscape horizons somewhat closer to the canonical upright orientation. For fractal scenes, there was no signification head roll for any of the scene rotations.

Collectively, these data for both head and eye reveal that both systems are affected by the content of the 360° panorama for this particular task. For landscape scenes, both effector systems seek out the horizon, even when the images are rotated. And when these scenes are rotated, the head often rotates to return the landscape horizons closer to their canonical upright orientation. For fractals, which have an isotropic texture, the behavior of the eye and head systems change dramatically, but in a similar way to one another. They are both insensitive to image rotation and display a preferential bias to move in the world-centered cardinal coordinates, with the greatest amplitude being horizontal. This finding dovetails with work in more naturalistic contexts, where eye and head movements show a strong bias to move in the cardinal directions, but that the relative contribution of vertical and horizonal movements depends on the particular environment (e.g., train station, forest, or apartment [Einhäuser et al., 2007]; navigating routes of various complexity [‘t Hart & Einhäuser, 2012]). Understanding how scene type and task differences (looking vs. navigating) may influence gaze and head movement biases in virtual environments is likely to be a fruitful avenue for future research. What these data show unequivocally, is that saccade biases previously observed are not merely an artefact of laboratory-based eye tracking studies, limited to situations where head movements are discouraged or prohibited. Indeed, it appears that when the head movements are permitted, they tend to follow and operate in service of the eyes (e.g., rotating the head to enable horizontal eye movements).

The analysis of the relationship between gaze and head movements indicates that while the two movements are similar, their functional relationship appears to be complementary. An analysis of the distance between gaze and head positions revealed that gaze and head positions coincided quite closely, but at a temporal lag. Specifically, gaze lead the head by about 200 ms, consistent with earlier findings where such dynamics indicated unplanned, reflexive movements (see e.g., Pelz et al., 2001; Freedman, 2008; Doshi & Trivedi, 2012). Finally, an analysis of eye-in-head position revealed that the eye stayed relatively close to the center of the head, consistent with the observations of Foulsham et al. (2011) who found that most eye movements occurred, relative to the head, within the center of the visual field (see also Foulsham & Kingstone, 2017). Interestingly, this was also sensitive to image content and rotation (Figure 18). This finding suggests that gaze and head have complementary roles in visual exploration, confirming similar findings from Fang, Nakashima, Matsumiya, Kuriki, and Shioiri (2015) and Solman et al. (2017).

Throughout the analysis of gaze and head behavior, it was clear that there were significant individual differences in gaze and head exploration, as well as head rotation, or roll (Figures 8, 14 and 15). Past work with the head restrained has demonstrated that there are indeed idiosyncratic tendencies in eye movement behavior (e.g., Foulsham & Underwood, 2008; Tatler & Vincent, 2008; Foulsham et al., 2014), and in scene exploration (Risko, Anderson, Lanthier & Kingstone, 2012). In terms of head movements, there was early indication that some participants, when left to their own devices, would move their heads to targets via audio or simple light display cues, whereas others preferred to move only their eyes (Delreux, Abeele, Lefevre & Roucoux, 1991; Fuller, 1992a, 1992b; Pozzo, Berthoz & Lefort, 1992; Goldring, Dorris, Corneil, Ballantyne & Munoz, 1996; Stahl, 2001). Fuller (1992b) dubbed his participants as either “movers” or “nonmovers,” but noted that the differences among them varied along a continuous spectrum. This finding dovetails with our own observations, both in the VR lab more generally, and in the data presented here. Fuller suggests that the differences may result from differing uses of egocentric versus allocentric reference frames, but so far, most reports of this idiosyncratic behavior are observational in nature, and reported alongside other experimental work.

### Implications for visual exploration

In everyday life, we rely on visual input for a multitude of reasons, such as to detect persons and objects of interest and to interact with them, to plan navigational paths through the environment and execute these plans while avoiding obstacles, to recognize and respond to potentially dangerous events, and to explore and remember the structure of the environment around us. The present study is focused exclusively on the last aspect, namely, the visual exploration of the environment. To this effect, we studied basic exploration characteristics in a VR environment, which allows one to not only have full control over the environment that observers are exploring, but to also study how their head and eye movement systems are interacting during their exploration. To our knowledge this is the first study to systematically investigate the relationship between eye and head movements while observers view fully immersive panoramic scenes. We have found that, when people are free to explore these scenes, they move both their head and their eyes. Although this finding may raise concerns for past work that has focused almost exclusively on head-fixed viewing, we found that head movements closely mirror eye movement behavior. Yet, the head movement data reveal significant, and new findings. First, the head seems to act in service of the eye. Not only does it follow the eye to keep the eye centered in the head, it rotates to bring the landscape images into a more canonical upright position for exploration. Second, dynamic patterns of eye and head movements that differ between landscape and fractal images reveal specific scanning strategies that are exquisitely sensitive to the scene content.

As discussed elsewhere in this article, there are several limitations to our work. First, the panoramic scenes were static and presented without binocular or kinematic depth cues to keep our system and analysis sufficiently simple. Second, our study does not take the contribution of torsional eye movements into account, which we were unable to measure using our system. Third, by determining head positions with respect to fixation positions, we average over much of the dynamics of head movement behavior, which is inherently smoother and slower than gaze behavior. Clearly, there are many future opportunities for research in VR (see for example Diaz, Cooper, Kit & Hayhoe, 2013; Fang et al., 2015; Weber, Schubert, Vogt, Velichkovsky & Pannasch, 2018), and this is particularly exciting as virtual environments become more realistic and movement in VR becomes more natural.

Our results demonstrate that a VR environment constitutes a very useful and informative alternative to other approaches to eye movement research, to those methods that use static and dynamic scenes presented on a monitor, and to those that investigate active exploration using mobile eye trackers during navigation through, or manipulation of real or virtual environments. The present study bridges those two classes of studies, and the outlook for future studies with static and dynamic VR environments looks very promising indeed.

## Figures and Tables

**Figure A1.**
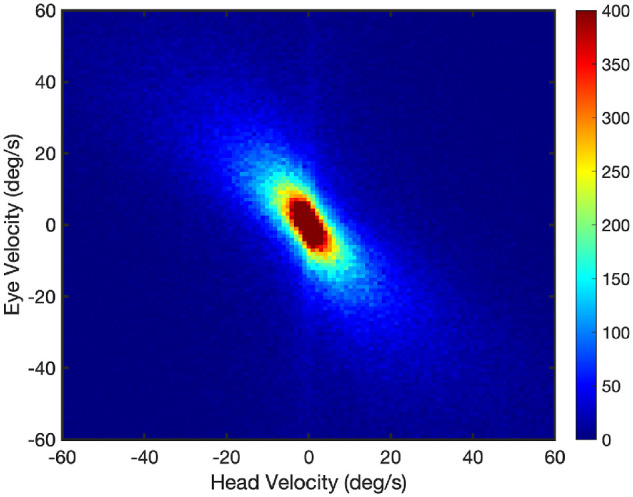
VOR demonstrated by the inverse relationship between eye and head velocities, using a bin size of 1°/s. Dark blue pixels correspond to zero frequency counts, and dark red pixels correspond to a bin count of more than 400.

## Appendix A: Circular statistics

The gaze tracker values were transformed into longitude and latitude of the spherical panorama, with longitude in the range of −180° to 180° and latitude in the range of −90° to 90°. To cope with the wrap-around at ±180° longitude, means and dispersion of longitude values were computed using circular statistics (Batschelet, 1981; Mardia & Jupp, 2000).

### Circular mean

Given a set of longitude values *λ_1,_ λ_2__, …,_ λ_n_* in the range of −180° to 180°, the mean longitude *L* is defined as

$$L\;=\;\tan^{-1}\left(\sum_{i\;=\;1}^{n}\sin\lambda_{i}/\sum_{i\;=\;1}^{n}\cos\lambda_{i}\right)$$

### Circular range

Given a set of longitude values *λ_1,_ λ_2__, …,_ λ_n_* in the range of −180° to 180°, the range *R* is defined as follows: Let *λ_(1),_ λ_(2), …,_ λ_(n)_* be the linearly ordered *λ_1,_ λ_2, …,_ λ_n_*. Define *T_i =_ λ_(i+1_**_)_ –* *λ_(i)_* for *i = 1, …, n* *–* *1* and *T_n_ =* 360 − *λ_(n)_* + *λ_(1)_*. Then the circular range *ω* is defined as

$$\omega \;=\;360-\max\left(T_{1},T_{2},...,T_{n}\right).$$

Saccades are defined as differences between successive fixations, more precisely in terms of great circles on the spherical panorama: Given are two fixations *f_1_ = (λ_1_, φ_1_)* and *f_2_ = (λ_2_, φ_2_)* with longitudes *λ_1_, λ_2_* and latitudes *φ_1_, φ_2_*.

The *saccade amplitude* between fixations *f_1_* and *f_2_* is defined by the great-circle distance

$$\Delta \sigma \;\;\;\;=\;\;\;r\cdot \cos^{-1}\left(\sin\;\varphi_{1}\sin\;\varphi_{2}\;+\;\cos\varphi_{1}\cos\;\varphi_{2}2\cos\;\left|\Delta \;\lambda \;\right|\right),$$

where Δλ  = λ_2_  − λ_1_ and r is the sphere radius.

The *saccade direction α_1_* starting at fixation *f_1_* is defined by the azimuth of the great circle at *f_1_*, is defined by

$$\begin{array}{c} \begin{array}{c} \;\alpha_{1}=\tan^{-1}(\cos\varphi 2\sin\Delta \lambda /\cos\varphi 1\sin\varphi 2\\ \;\;\;\;-\sin\varphi 1\cos\varphi 2\cos\Delta \lambda ) \end{array} \end{array}$$

## Appendix B: Gaze–head lag analysis

Give are two sequences, a sequence *G = g_1_ g_2_ … g_n_* of gaze positions and a sequence *H = h_1_ h_2_ … h_n_* of head positions. The calculation of the head position sequence H is described in section on gaze–head lag. The minimum distance method is based on calculating all *g_i_* *–* *h_j_* distances within a fixed temporal window of size [–*k,k*]. We used *k* = 8 for our analyses. For each gaze direction *g_i_* and a set of head positions *h_j_* with *j = i**–**k, i**–(**k**–**1), …, i**–**1, i, i+1, …, i+(k**–**1), i+k*, the pair *g_i_* *–* *h_j_* with minimum distance is determined. If *i < j*, that is, the *h_j_* with minimum distance occurred before *g_i_*_,_ then it was concluded that head was leading gaze, otherwise if *i > j*, that is, *h_j_* with minimum distance occurred after *g_i_*, then it was concluded that head was lagging behind gaze.

## Appendix C: Vestibulo-ocular reflex

We have also identified the VOR (Barnes, 1979; Laurutis & Robinson 1986) in our data. This reflexive mechanism moves the eyes in the opposite direction of the head movement, to stabilize the perceptual input. Participants differed substantially with respect to the proportion of very slow head movements (with an absolute velocity |*v*|  ≤  2°/*sec*), ranging from 10.5% to 57.7% of the velocity measurements. Given that very slow head movements cannot be used to investigate the VOR, participants with 25% or more slow head movements were excluded from the VOR analysis.

Figure A1 shows the relationship between longitudinal head and eye velocities (i.e., the figure is based on the longitudinal components of the head and eye vectors). The raw eye and head data were sampled at approximately 50 Hz, and the figure is thus based on approximately 380,000 data points. As the figure shows, the data points cluster along the inverse linear relationship between head and eye velocities, as expected from the VOR.

## References

[bib1] Anderson N. C., Bischof W. F., Foulsham T., & Kingstone A. (in press). Turning the (virtual) world around: Patterns in saccade direction vary with picture orientation and shape in virtual reality. *Journal of Vision*. 10.1167/jov.20.8.21 PMC7443121 38755788

[bib2] Barnes G. R. (1979). Vestibulo-ocular function during co-ordinated head and eye movements to acquire visual targets. *Journal of Physiology,* 287, 127–147. 311828 10.1113/jphysiol.1979.sp012650 PMC1281486

[bib3] Batschelet E. (1981). *Circular statistics in biology*. London: Academic Press.

[bib4] Birmingham E., Bischof W. F., & Kingstone A. (2008). Gaze selection in complex social scenes. *Visual Cognition,* 16, 341–355, 10.1080/13506280701434532.

[bib5] Birmingham E., Bischof W. F., & Kingstone A. (2009). Saliency does not account for fixations to eyes within social scenes. *Vision Research,* 49, 2992–3000, 10.1016/j.visres.2009.09.014. 19782100

[bib6] Bizzi E., Kalil R. E., & Tagliasco V. (1971). Eye-head coordination in monkeys: Evidence for centrally patterned organization. *Science,* 173, 452–454. 17770450 10.1126/science.173.3995.452

[bib7] Blignaut P. (2009). Fixation identification: The optimum threshold for a dispersion algorithm. *Attention, Perception, & Psychophysics,* 71(4), 88–895, 10.3758/APP.71.4.881. 19429966

[bib8] Buswell G. T. (1935). *How people look at pictures: A study of the psychology of perception in art*. Chicago, IL: University of Chicago Press.

[bib9] Chandrakumar M., Blakeman A., Goltz H. C., Sharpe J. A., & Wong A. M. F (2011). Static ocular counterroll reflex in skew deviation. *Neurology,* 77, 638–644, 10.1212/WNL.0b013e3182299f71. 21813791 PMC3159094

[bib10] Coppola D. M., Purves H. R., McCoy A. N., & Purves D. (1998). The distribution of oriented contours in the real world. *Proceedings of the National Academy of Sciences of the United States of American,* 95(7), 4002–4006, 10.1073/pnas.95.7.4002. PMC19952 9520482

[bib11] Cristino F., & Baddeley R. (2009). The nature of visual representations involved in eye movements when walking down the street. *Visual Cognition,* 17(6–7), 880–903, https://doi.org/0.1080/13506280902834696.

[bib12] Delreux V., Abeele S. V., Lefevre P., & Roucoux A. (1991). Eye–head coordination: Influence of eye position on the control of head movement amplitude. In J Paillard (Ed.), *Brain and space* (pp. 38–48). London: Oxford University Press.

[bib13] Diaz G., Cooper J., Kit D., & Hayhoe M. (2013). Real-time recording and classification of eye movements in an immersive virtual environment. *Journal of Vision,* 13(12), 5–5, 10.1167/13.12.5. [Article] PMC3795427 24113087

[bib14] Doshi A., & Trivedi M. M. (2012). Head and gaze dynamics during visual attention shifts in complex environments. *Journal of Vision,* 12(2), 1–15, 10.1167/12.2.9. [Article] 22323822

[bib15] Einhäuser W., Schumann F., Bardins S., Bartl K., Böning G., Schneider E., & König P. (2007). Human eye-head co-ordination in natural exploration. *Network: Computation in Neural Systems,* 18(3), 267–297, https://doi.org/0.1080/09548980701671094. 10.1080/09548980701671094 17926195

[bib16] Fang Y., Nakashima R., Matsumiya K., Kuriki I, & Shioiri S. (2015) Eye-head coordination for visual cognitive processing. *PLoS One,* 10 (3), e0121035, 10.1371/journal.pone.0121035. [10.1371/journal.pone.0121035] 25799510 PMC4370616

[bib17] Foulsham T., Chapman C., Nasiopoulos E., & Kingstone A. (2014). Top-down and bottom-up aspects of active search in a real world environment. *Canadian Journal of Experimental Psychology,* 68, 8–19, 10.1037/cep0000004. 24219246

[bib18] Foulsham T., Cheng J. T., Tracy J. L, Henrich J., & Kingstone A. (2010). Gaze allocation in a dynamic situation: Effects of social status and speaking. *Cognition,* 117, 319–331, 10.1016/j.cognition.2010.09.003. 20965502

[bib19] Foulsham T., & Kingstone A. (2010). Asymmetries in the direction of saccades during perception of scenes and fractals: Effects of image type and image features. *Vision Research,* 50, 779–795, 10.1016/j.visres.2010.01.019. 20144645

[bib20] Foulsham T., & Kingstone A. (2017). Are fixations in static natural scenes a useful predictor of attention in the real world? *Canadian Journal of Experimental Psychology,* 71, 172–181, 10.1037/cep0000125. 28604053

[bib21] Foulsham T., Kingstone A., & Underwood G. (2008). Turning the world around: Patterns in saccade direction vary with picture orientation. *Vision Research,* 48, 1777–1790, 10.1016/j.visres.2008.05.018. 18599105

[bib22] Foulsham T., & Underwood G. (2008). What can saliency models predict about eye movements? Spatial and sequential aspects of fixations during encoding and recognition. *Journal of Vision,* 8(2), 6–6, 10.1167/8.2.6. [10.1167/8.2.6] 18318632

[bib23] Foulsham T., Walker E., & Kingstone A. (2011). The where, what and when of gaze allocation in the lab and the natural environment. *Vision Research,* 51, 1920–1931, 10.10.1016/j.visres.2011.07.002. 21784095

[bib24] Freedman E. G. (2008). Coordination of the eyes and head during visual orienting. *Experimental Brain Research,* 190, 369–387, 10.1007/s00221-008-1504-8. 18704387 PMC2605952

[bib25] Fuller J. (1992a). Comparison of head movement strategies among mammals. In Berthoz A. Graf W. Vidal P. P. (Eds.), *The head-neck sensory motor system* (pp. 101–112). Oxford: Oxford University Press, 10.1093/acprof:oso/9780195068207.003.0013.

[bib26] Fuller J. (1992b). Head movement propensity. *Experimental Brain Research,* 92(1), 152–164. 1486950 10.1007/BF00230391

[bib27] Gilchrist I. D., Brown V., Findlay J. M., & Clarke M. P. (1998). Using the eye–movement system to control the head. *Proceedings of the Royal Society of London B,* 265, 1831–1836, https://doi.org/ 10.1098/rspb.1998.0509. 10.1098/rspb.1998.0509 PMC1689378 9802239

[bib28] Goldring J. E., Dorris M. C., Corneil B. D., Ballantyne P. A., & Munoz D. R. (1996). Combined eye-head gaze shifts to visual and auditory targets in humans. *Experimental Brain Research,* 111(1), 68–78, 10.1007/BF00229557. 8891638

[bib29] Hayhoe M., & Ballard D. (2005). Eye movements in natural behavior. Trends in Cognitive Sciences, 9(4), 188–194, 10.1016/j.tics.2005.02.009. 15808501

[bib30] Hessels R. S., Niehorster D. C., Nyström M., Andersson R., & Hooge I. T. C. (2018). Is the eye-movement field confused about fixations and saccades? A survey among 124 researchers. *Royal Society Open Science**,* 5, 180502, 10.1098/rsos.180502. [10.1098/rsos.180502] 30225041 PMC6124022

[bib31] Kingstone A., Smilek D., & Eastwood J. D. (2008). Cognitive ethology: A new approach for studying human cognition. *British Journal of Psychology,* 99(3), 317–340, 10.1348/000712607X251243. 17977481

[bib32] Komogortsev O. V., Gobert D. V., Jayarathna S., Koh D., & Gowda S. (2010). Standardization of automated analyses of oculomotor fixation and saccadic behaviors. *IEEE Transactions on Biomedical Engineering,* 57(11), 2635–2645, 10.1109/TBME.2010.2057429. 20667803

[bib33] Land M. F., & Hayhoe M. (2001). In what ways do eye movements contribute to everyday activities? *Vision Research,* 41(25–26), 3559–3565, 10.1016/S0042-6989(01)00102-X. 11718795

[bib34] Land M. F., Mennie N., & Rusted J. (1999). The roles of vision and eye movements in the control of activities of daily living. *Perception,* 28, 1311–1328, 10.1068/p2935. 10755142

[bib35] Land M. F., & Tatler B. W. (2009). *Looking and acting: Vision and eye movements in natural behaviour*. Oxford, UK: Oxford University Press, 10.1093/acprof:oso/9780198570943.001.0001.

[bib36] Laurutis V., & Robinson D. (1986). The vestibulo-ocular reflex during human saccadic eye movements. *Journal of Physiology,* 373, 209–33, 10.1113/jphysiol.1986.sp016043. 3489091 PMC1182533

[bib37] Mackworth N. H., & Morandi A. J. (1967). The gaze selects informative details within pictures. *Perception & Psychophysics,* 2, 547–552.

[bib38] Mardia K. V., & Jupp P. E. (2000). *Directional statistics*. Chichester: Wiley.

[bib39] Mathworks (2019). Matlab 2019a [Computer Software], https://www.mathworks.com/.

[bib40] Morasso P., Sandini G., Tagliasco V., & Zaccaria R. (1977). Control strategies in the eye–head coordination system. *IEEE Transactions on Systems, Man and Cybernetics,* 7, 639–651.

[bib41] Niehorster D. C., Li L., & Lappe M. (2017). The accuracy and precision of position and orientation tracking in the HTC Vive virtual reality system for scientific research. *I-Perception,* 8(3), 1–23, 10.1177/2041669517708205. [10.1177/2041669517708205] PMC5439658 28567271

[bib42] Parkhurst D., Law K., Niebur E (2002). Modeling the role of salience in the allocation of overt visual attention. *Vision Research,* 42(1), 107–123, 10.1016/S0042-6989(01)00250-4. 11804636

[bib43] Pelz J., Hayhoe M., & Loeber R. (2001). The coordination of eye, head, and hand movements in a natural task. *Experimental Brain Research,* 139(3), 266–277, 10.1007/s002210100745. 11545465

[bib44] Potter M. C., Staub A., & O'Connor D. H. (2004). Pictorial and conceptual representation of glimpsed pictures. *Journal of Experimental Psychology – Human Perception and Performance,* 30(3), 478–489, 10.1037/0096-1523.30.3.478. 15161380

[bib45] Pozzo T., Berthoz A., & Lefort L. (1992). Head kinematics during complex movements. In Berthoz A. Graf W. Vidal P. P. (Eds.), *The head-neck sensory motor system* (pp. 587–590). Oxford: Oxford University Press, 10.1093/acprof:oso/9780195068207.003.0095.

[bib46] Risko E. F., Anderson N. C., Lanthier S., & Kingstone A. (2012). Curious eyes: Individual differences in personality predict eye movement behavior in scene-viewing. *Cognition,* 122(1), 86–90, 10.1016/j.cognition.2011.08.014. 21983424

[bib47] Salvucci D. D., & Goldberg J. H. (2000). Identifying fixations and saccades in eye-tracking protocols. In *Proceedings of the EyeTracking Research and Applications Symposium* (pp. 71–78). New York: ACM Press, 10.1145/355017.355028.

[bib48] Schizo604 (2010). Descent into fractal core and fractal matrix [Video]. Retrieved from http://www.wearvr.com/apps/fractal-canyon-vr.

[bib49] Schmid-Priscoveanu A., Straumann D., & Kori A. A. (2000). Torsional vestibulo-ocular reflex during whole-body oscillation in the upright and the supine position. I. Responses in healthy human subjects. *Experimental Brain Research,* 134, 2011–2019, 10.1007/s002210000436. 11037288

[bib50] Schor C. M. (2011). Neural control of eye movements. In Levin L. A. Nilsson SFE S. F. E. Ver Hoeve J. Wu SM S. M. (Eds.), *Adler's physiology of the eye* (11th ed., pp. 220–242). Philadelphia: Saunders/Elsevier, 10.1016/B978-0-323-05714-1.00009-1.

[bib51] SensoMotoric. (2017). SensoMotoric Instruments. [Apparatus and software]. https://en.wikipedia.org/wiki/SensoMotoric_Instruments.

[bib52] Sitzmann V., Serrano A., Pavel A., Agrawala M., Gutiérrez D., Masia B., & Wetzstein G. (2018). Saliency in VR: How do people explore virtual environments? *IEEE Transactions on Visualization and Computer Graphics,* 24(4), 1633–1642, 10.1109/TVCG.2018.2793599. 29553930

[bib53] Solman G. J. F., Foulsham T., & Kingstone A. (2017). Eye and head movements are complementary in visual selection. *Royal Society Open Science,* 4, 160569, 10.1098/rsos.160569. [10.1098/rsos.160569] 28280554 PMC5319320

[bib54] Solman G. J. F., & Kingstone A. (2014). Balancing energetic and cognitive resources: Memory use during search depends on the orienting effector. *Cognition,* 132, 443–454, 10.1016/j.cognition.2014.05.005. 24946208

[bib55] Stahl J. S. (2001). Eye-head coordination and the variation of eye-movement accuracy with orbital eccentricity. *Experimental Brain Research,* 136(2), 200–210, 10.1007/s002210000593. 11206282

[bib56] Stata. (2019). Stata 15.1 [Computer software], https://www.stata.com.

[bib57] Tatler B. W. (2007) The central fixation bias in scene viewing: Selecting an optimal viewing position independently of motor biases and image feature distributions. *Journal of Vision,* 7(14), 4, 1–17, 10.1167/7.14.4. [Article] 18217799

[bib58] Tatler B. W., Hayhoe M. M., Land M. F., & Ballard D. H. (2011). Eye guidance in natural vision: Reinterpreting salience. *Journal of Vision,* 11(5), 5, 10.1167/11.5.5. [Article] PMC3134223 21622729

[bib59] Tatler B. W., & Vincent B. T. (2008). Systematic tendencies in scene viewing. *Journal of Eye Movement Research,* 2(2): 5, 1–18, 10.16910/jemr.2.2.5. [10.16910/jemr.2.2.5]

[bib60] Technologies U. (2015). Unity - Manual: Unity Manual. [online] Docs.unity3d.com. Retrieved from https://docs.unity3d.com/Manual/index.html.

[bib61] ’t Hart B. M., Vockeroth J., Schumann F., Bartl K., Schneider E., König P., & Einhäuser W. (2009). Gaze allocation in natural stimuli: Comparing free exploration to head-fixed viewing conditions. *Visual Cognition,* 17(6–7), 1132–1158, 10.1080/13506280902812304.

[bib62] ’t Hart B. M., & Einhäuser W. (2012). Mind the step: Complementary effects of an implicit task on eye and head movements in real-life gaze allocation. *Experimental Brain Research,* 223(2), 233–249, 10.1007/s00221-012-3254-x. 23001370

[bib63] Torralba A. (2003). Modeling global scene factors in attention. *Journal of the Optical Society of America A – Optics Image Science and Vision,* 20(7), 1407–1418, https://doi.org/0.1364/JOSAA.20.001407. 10.1364/josaa.20.001407 12868645

[bib64] Underwood G., & Foulsham T. (2006). Visual saliency and semantic incongruency influence eye movements when inspecting pictures. *Quarterly Journal of Experimental Psychology,* 59(11), 1931–1949, 10.1080/17470210500416342. 16987782

[bib65] Underwood G., Foulsham T., Humphrey K., (2009). Saliency and scan patterns in the inspection of real-world scenes: Eye movements during encoding and recognition. *Visual Cognition,* 17(6–7), 812–834, 10.1080/13506280902771278.

[bib66] Unema P. J. A., Pannasch S., Joos M., & Velichkovsky B. M. (2005). Time course of information processing during scene perception: The relationship between saccade amplitude and fixation duration. *Visual Cognition,* 12(3), 473–494, 10.1080/13506280444000409.

[bib67] Weber S., Schubert R. S., Vogt S., Velichkovsky B. M., & Pannasch S. (2018). Gaze3DFix: Detecting 3D fixations with an ellipsoidal bounding volume. *Behavior Research Methods,* 50(5), 2004–2015, 10.3758/s13428-017-0969-4. 29076105

[bib68] Wikipedia. (2019). Equirectangular projection, https://en.wikipedia.org/wiki/Equirectangular_projection.

[bib69] Xiao J., Ehinger A., Oliva A., & Torralba A. (2012). Recognizing scene viewpoint using panoramic place representation. *2012 IEEE Conference on Computer Vision and Pattern Recognition,* 2012, 2695–2702, 10.1109/CVPR.2012.6247991.

[bib70] Yarbus A. L (1967). *Eye movements and vision* (Haigh B., Trans.). New York: Plenum Press. (Original work published 1965)

